# Rational Design and Organoid‐Based Evaluation of a Cocktail CAR‐γδ T Cell Therapy for Heterogeneous Glioblastoma

**DOI:** 10.1002/advs.202501772

**Published:** 2025-03-20

**Authors:** Guidong Zhu, Zhongzheng Sun, Yingchao Liu, Jiang Liu, Linpei Guo, Guojing Pei, Ying Jiang, Baowang Miao, Zhen Li, Ping Zhang, Dongqi Tang, Wen Zhang, Chengwei Wang

**Affiliations:** ^1^ Department of Neurosurgery, The Second Hospital, Cheeloo College of Medicine Shandong University Jinan 250031 P.R. China; ^2^ Department of Neurosurgery Provincial Hospital Affiliated to Shandong First Medical University Jinan 250031 P.R. China; ^3^ Institute of Medical Sciences, The Second Hospital, Cheeloo College of Medicine Shandong University Jinan 250031 P.R. China; ^4^ Multidisciplinary Innovation Center for Nephrology of the Second Hospital of Shandong University Jinan 250031 China

**Keywords:** cocktail therapy, glioblastoma, multiple CAR‐γδ T, organoid, personalized strategy

## Abstract

Various challenges, including tumor heterogeneity and inadequate T cell infiltration, impede the progress of chimeric antigen receptor T cell (CAR‐T) therapy for glioblastoma (GBM). To address these obstacles, a multiple step strategy is designed. Initially, literature review and bioinformatics analysis to screen a set of antigens that are heterogeneously expressed in GBM, which are designated as the target‐bank, are leveraged. Then, according to the multiplex immunohistochemistry results of each patient's tumor sample, a personalized panel of antigens based on the principle that most cancer cells in tumor tissues can be covered from the target‐bank is selected. To target these antigens, Vδ1 T cells are chosen as CAR vehicles because of its high tissue infiltration and off‐the‐shelf properties, and an optimized protocol for engineering CAR‐Vδ1 T cells with high purity and cytotoxicity, low exhaustion, and cytokine release is developed. Next, the specific panel of cocktail CAR‐Vδ1 T cells in the GBM organoids that are directly derived from the same patient's tumor is tested. The term “***prof***” cocktail therapy is coined to describe the approach using precise and rational combination of tumor antigens, organoid‐based evaluation, and fitness of Vδ1 T cells. It may accelerate development of effective CAR‐T drugs for heterogeneous solid tumors.

## Graphical abstract

A novel “***prof*** *”* cocktail therapy is designed. It screens antigens, selects personalized antigen panels, engineers optimized CAR‐Vδ1 T cells, and tests in patient‐derived GBM organoids, offering hope for effective CAR‐T drugs against heterogeneous solid tumors.

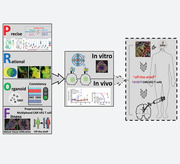

## 1 Introduction

Chimeric antigen receptor T (CAR‐T) cells therapy have achieved great deal of success in hematologic malignancies.^[^ ^1^ ^]^ However, it is not widely used for treating solid tumors due to their poor efficacy.^[^ ^2^, ^3^, ^4^, ^5^ ^]^ Glioblastoma (GBM) is the most invasive and lethal glioma and presents a formidable challenge in the development of effective treatment strategies.^[^ ^6^, ^7^ ^]^ CAR‐T cell treatment can cause tumor regression in preclinical GBM models;^[^ ^8^, ^9^, ^10^ ^]^ however, clinical trials have only yielded sporadic clinical responses.^[^ ^11^, ^12^, ^13^, ^14^ ^]^ One reason for this may be the high cell heterogeneity of GBM. Due to the heterogeneity of tumors cells, the intrinsic specific targeting property of CAR‐T cells has become a disadvantageous factor, and the residual cells would cause recurrence even the dominant target of tumor cells are killed. In addition, the tumor microenvironment (TME) exacerbates GBM growth, suppresses infiltration and kill function of CAR‐T cell.^[^ ^2^, ^3^, ^4^ ^]^ Thus, multiple factors may hamper the efficacy of CAR‐T cells in solid tumors, and developing a comprehensive strategy to overcome these obstacles is critical.

Using a multiple CAR‐T cell approach may overcome solid tumor heterogeneity. Clinical trials utilizing single‐target CAR‐T cells, such as those directed against IL13Rα2, HER2, and EGFRvIII, have demonstrated limited efficacy in the treatment of GBM.^[^ ^11^, ^12^, ^13^, ^14^ ^]^ Dual‐specific CARs targeting the pan‐tumor antigens CD70 and B7‐H3 have shown considerable efficacy in multiple solid tumors.^[^ ^15^ ^]^ Nevertheless, complete tumor clearance could not be achieved in the latest clinical trials of EGFR and IL13Rα2 dual‐target CAR‐T cell therapy for GBM.^[^ ^16^ ^]^ Therefore, the precise and personalized selection of antigens as targets to cover most or even all the tumor cells is crucial while designing effective CAR‐T cell therapies.

T cell receptors (TCRs) comprise two receptor types: αβ and γδ chain receptors.^[^ ^17^ ^]^ Nowadays, most of CAR‐T cells are based on αβ T cells and are autologous, but they have disadvantages such as complex manufacturing processes, long cycles, and high prices. Furthermore, non‐off‐the‐shelf, limited tumor infiltration ability also restricts the application of CAR‐T cells in treating solid tumors. On the other hand, γδ T cells recognize several antigens on various tumor cells through different innate cytotoxic receptors.^[^ ^18^ ^]^ The non‐major histocompatibility complex‐restricted recognition manner of γδ T cells allows the development of universal γδ T cells suitable for ready‐to‐use applications. Notably, γδ T cells, especially the Vδ1 subset, naturally reside in various tissues and display inherent tissue infiltration, rapid response to targets, and effector cytokine release.^[^ ^19^, ^20^, ^21^, ^22^ ^]^ Moreover, γδ T cells exhibit higher infiltration capacity and functionality in low‐oxygen environments than αβ T cells.^[^ ^23^ ^]^ Therefore, using γδ T cells may represents an alternative or better strategy that could be more effective for treating solid tumors than αβ T cells.^[^ ^24^, ^25^, ^26^, ^27^ ^]^

**Scheme 1:**
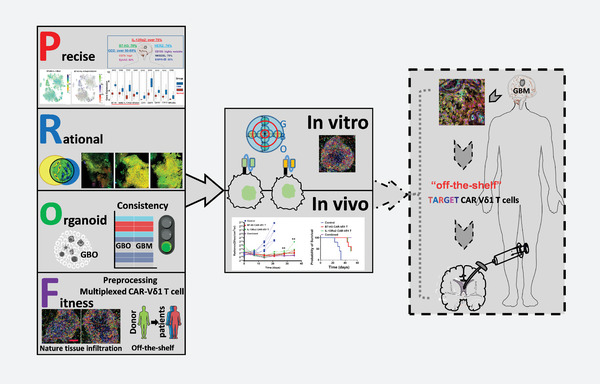
**“*Prof*”** multiple CAR‐ Vδ1T cell cocktail therapy schematic diagram.

It is of great advantage to know the efficacy before the infusion of CAR‐T cells. Owing to the complexity of solid tumors, accurately evaluating the efficacy of CAR‐T cells in vitro is challenging. Conventional drug screening models cannot predict the actual responses of patients to CAR‐T cell therapy.^[^ ^28^ ^]^ Tumor organoids derived from patient preserve critical structural and functional features of their in vivo counterparts, making them essential tools for developing personalized medicine. Importantly, dynamic 3D specialized cultivation techniques can mimic crucial aspects of the TME, including dynamic blood flow, cell–cell interactions, and the presence of immunosuppressive immune cells. This enables accurate predictions of patient responses to personalized cell‐based therapies.^[^ ^29^, ^30^, ^31^, ^32^ ^]^ Therefore, organoid technology can streamline the evaluation process and accelerate the development of multiple CAR‐T therapy. In this study, a multi‐step strategy for CAR‐Vδ1 T cell cocktail therapy was developed to treat heterogeneous GBM. The comprehensive approach using **p**recise antigen profiles and **r**ational combinations of tumor antigens, **o**rganoid‐based evaluation, and **f**itness of Vδ1 T cells, collectively termed the “***prof***” cocktail therapy, holds promise as a potential for treating heterogeneous solid tumors (**Scheme** **1**).

## 2 Results

### 2.1 Target‐Bank and Their Expression in GBM

**Figure 1:**
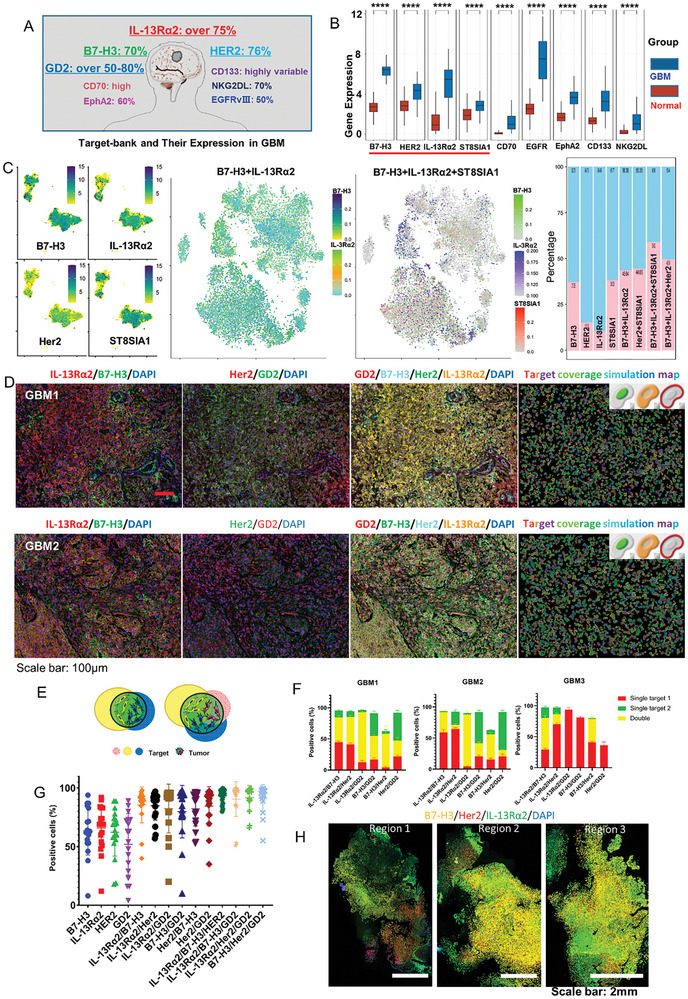
Heterogeneous B7‐H3, IL‐13Rα2, Her2, and GD2 expression in GBM and the broad spectrum of tumor cells covered by personalized dual or triple target combinations. A) Target‐bank and their characteristics. For detailed information, please refer to Table S1 (Supporting Information). B) The expression distribution of target gene in tumor tissues and normal tissues. ****p < 0.0001, *n* = 153. The statistical difference of two groups was compared through the Wilcox test. C) Analysis of public scRNA‐seq datasets reveals heterogeneous gene expression in GBM and indicates that the screened gene combinations can improve coverage of tumor cell populations. D) Representative images of co‐staining in GBM. The horizontally arranged images were all of the same sample and same field of view. The rightmost column was the simulation map of target coverage. Scale bar: 100 µm. E) Schematic representation of dual or triple target schemes covering the entire tumor area. F) Proportion of positive cells in representative GBM samples with pairwise combination schemes. Data are presented as the mean ± SD of triplicate wells. G) Proportion of positive cells in dual or triple target combinations relative to total cell count (*n* = 18). H) Heterogeneous B7‐H3, IL‐13Rα2, and Her2 in expression different regions. Scale bar: 2 mm.

Given the high cost of CAR‐T cell therapy and the heterogeneity of GBM, it is impractical to pre‐prepare CAR‐T cells targeting all possible antigens or to explore new targets for every GBM patient. However, it is feasible to provide individualized target combination schemes for each patient by leveraging existing targets. Based on this strategy and leveraging our previous CAR‐T cell research experience and literature review, this study first established a target‐bank (B7‐H3, IL‐13Rα2, Her2, GD2, CD133, NKG2DL, CD70, EphA2, and EGFR) and sorted out the expression levels and characteristics of each target (**Figure** **1**A and Table S1, Supporting Information). Subsequently, we combined the expression levels of these target genes (or related genes, such as ST8SIA1 for GD2) in GBM and normal tissues from the Cancer Genome Atlas (TCGA) database (Figure 1B) to identify B7‐H3, IL‐13Rα2, Her2, and GD2 as the target pool for this study (screening criteria: relatively stable protein expression, expression rate >70%, and relatively high gene expression levels).

### 2.2 Combining Heterogeneous Antigens Covers Most Tumor Tissues

Integration and clustering of single‐cell RNA sequencing (scRNA‐seq) data were conducted for subsequent analyses. The integrated scRNA‐seq datasets included GSE131928 (24131 cells from 28 patients, 7930 cells by SMART‐Seq2, and 16201 by 10×, 16646 malignant cells, Figure 1C), GSE138794 (12276 cells from 9 patients by 10×, 7981 malignant cells, Figure S1A,B, Supporting Information), and GSE140819 (5400 cells from 2 patients by 10×, 1696 malignant cells, Figure S1C,D, Supporting Information). After selecting the malignant cells in the samples (Figure S2, Supporting Information), the initial analysis focused on the expression levels of B7‐H3, IL‐13Rα2, Her2, and ST8SIA1 in different GBM samples. The results revealed significant spatial distribution and expression heterogeneity of these target genes. Subsequent analysis of two‐gene combinations indicated that optimizing different combinations could cover a broader spectrum of tumor cell populations. Three‐gene combination analysis further enhanced coverage of tumor cell populations. Combinations such as B7‐H3 and IL‐13Rα2, and the three‐gene combination of B7‐H3, IL‐13Rα2, and GD2 showed high coverage across most samples (Figure 1C and Figure S1A–D, Supporting Information). These findings provide a theoretical basis and application prospects for multiple CAR‐γδ T cell therapy targeting heterogeneous tumor populations.

Immunofluorescence staining was performed on 18 pathology‐confirmed GBM paraffin samples (IDH wild‐type, Table S2, Supporting Information) to investigate B7‐H3, IL‐13Rα2, Her2, and GD2 distribution and expression in GBM. These targets were heterogeneously expressed in different tumors (Figure 1D). B7‐H3, IL‐13Rα2, and Her2 expression levels were inconsistent within different regions even in the same tumor (Figure 1H). Notably, a single target was insufficient to cover most tissues (Figure 1D, G). B7‐H3, IL‐13Rα2, Her2, and GD2 were categorized into double‐ or triple‐target groups to investigate the coverage of different target combinations. The double‐target combination covered >80% of regions, whereas the triple‐target combination covered > 90% of regions (Figure 1G). However, the coverage may vary among different tumors for each type of combination. The double‐target B7‐H3 and IL‐13Rα2 combination was more concentrated (having the highest average) than other double‐target combination in 18 GBM samples (Figure 1E–G). These results indicated that high coverage of tumor tissue could be achieved for individual patients using a rational combination of antigens in the profile, thereby laying the foundation for designing effective multiple CAR T cell therapies to treat heterogeneous tumors.

### 2.3 GBM Organoids (GBOs) Exhibit the Biological Characteristics of Original Tumor Tissues

**Figure 2:**
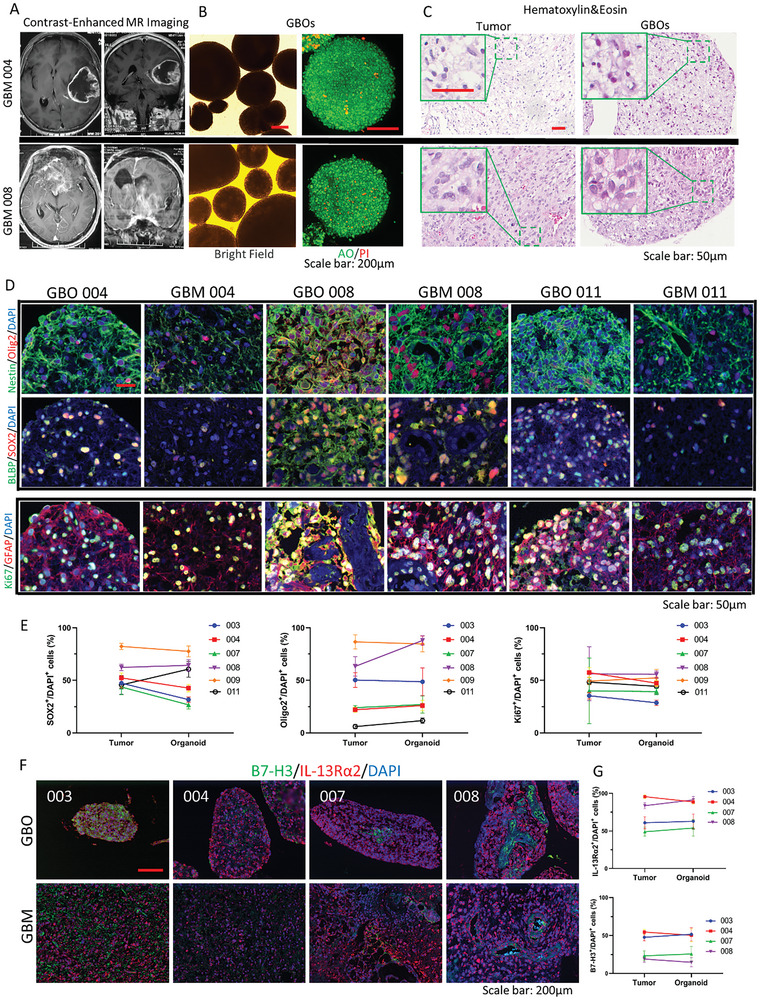
Cultivation and morphological and pathological identification of GBOs. A) Contrast‐enhanced MR imaging of the skull–brain for a patient with GBM. B) Bright‐field images and AO/PI staining results of representative circular organoids cultured from GBM tissue. Scale bar: 200 µm. C) Hematoxylin and Eosin Staining images of original tumor and corresponding GBO samples. Scale bar: 50 µm. D) Sample images of mIHC staining showing that cultured GBOs maintain cell populations of the corresponding original tumor tissues. Scale bar: 50 µm. E) Quantitative detection of SOX2^+^, OLIG2^+^, and Ki67^+^ cells in six original tumor tissues and corresponding GBOs. Data are presented as the mean ± SD of triplicate wells. F,G) Immunofluorescence displaying the B7‐H3 and IL‐13Rα2 expression in original tumor tissue and corresponding GBOs, with quantification. The data are presented as the mean ± SD of triplicate wells.

A novel culture method for GBO based on the literature^[^ ^33^ ^]^ was developed to validate the efficacy of multiple CAR‐T cell cocktail immunotherapies in relatively authentic patient tumor tissues. Fresh GBM samples were obtained from surgically excised tissues (**Figure** **2**A) and seeded with the GBO culture medium. After postoperative pathological confirmation, GBOs were successfully cultivated from 23 out of 26 GBM tissue samples, achieving an overall success rate of 88.46%. Tumor fragments typically formed round organoids within 1–2 weeks (Figure 2B). Acridine orange/propidium Iodide (AO/PI) staining revealed that the percentage of dead cells in the organoids was <10% (Figure 2B). Single GBOs were regularly observed and measured, revealing a gradual increase in size (Figure S3A, Supporting Information). Growth analysis of the six GBOs demonstrated that most tissue fragments formed independent GBOs (Figure S3B, Supporting Information).

The pathological features of high‐grade gliomas were confirmed using Hematoxylin and Eosin Staining to assess whether the GBOs resembled their corresponding parental tumors. The results indicated that GBOs exhibited cell and nuclear atypia similar to those of the original patient tumors (Figure 2C). Notably, some GBOs retained structures resembling the microvasculature (Figure 2D, GBO 008).

Multi‐fluorescence immunohistochemical analyses were conducted that included glial markers (GFAP), neural stem cells, GBM stem cell markers (Nestin, BLBP, SOX2, and OLIG2), and the Ki67 proliferation marker to determine the biological characteristics of GBOs further. GBOs displayed significant inter‐tumor heterogeneity (Figure 2D). The percentages of SOX2‐, OLIG2‐, and Ki67‐positive cells in the six tumor specimens demonstrated that this heterogeneity was highly similar to that of the corresponding parental tumors (Figure 2E).

Tumor inter‐ and intra‐heterogeneity was confirmed for target gene expression (B7‐H3, IL‐13Rα2) in corresponding GBOs through immunofluorescence (Figure 2F,G). These results suggested that GBOs inherit major molecular features of the original tumor tissue.

### 2.4 Whole Exome Sequencing Reveals Mutation Heterogeneity Maintenance in GBOs

**Figure 3:**
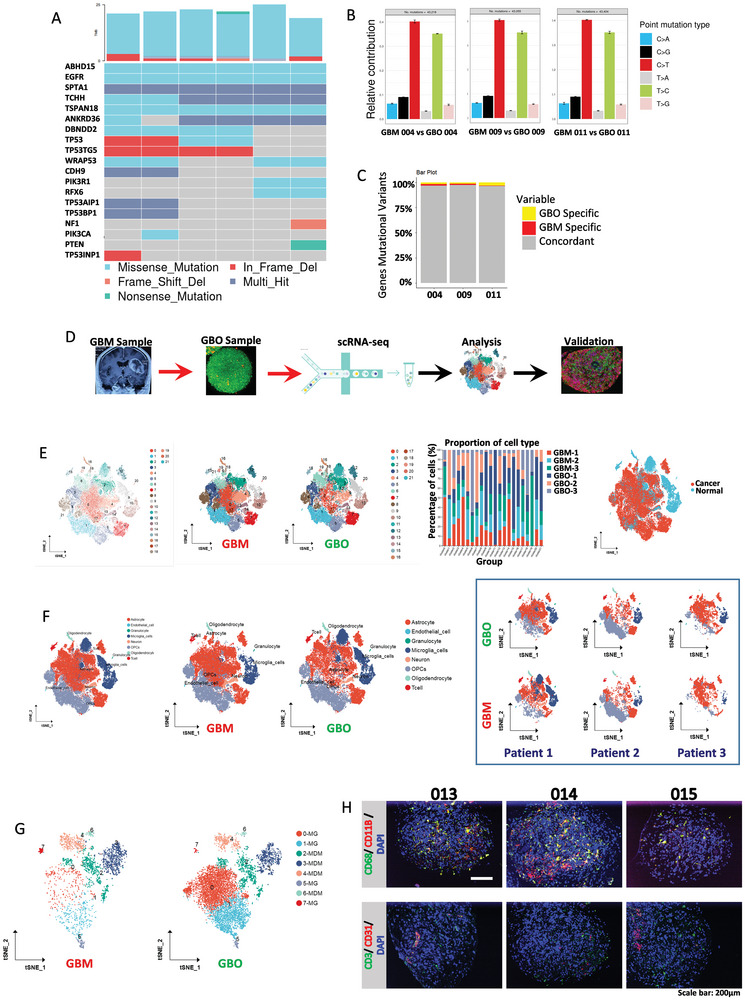
Revealing the consistency between GBOs and original tumor tissues through whole‐exome sequencing and scRNA‐seq. A) Landscape of mutated genes displaying the consistency of somatic cell variations between GBOs and parental GBM‐associated genes. B) Box plot of the total number and distribution of type point mutations demonstrating consistency between GBOs and parental GBM. C) Bar chart showing the number of shared and private mutations between GBOs and parental GBM. D) Flowchart of the scRNA‐seq process for GBOs. E) The t‐SNE plot shows the clustering and coloring of different cell clusters in GBO samples and their original GBMs, as well as the proportion of different cell clusters in different samples. The overall normal cell and malignant tumor cell clusters in six samples. F) The t‐SNE plot shows the clustering and coloring of different subsets in GBO and original GBM samples. G) The t‐SNE plot shows the re‐clustering and coloring of tumor‐associated macrophage subsets in GBO and original GBM samples. H) Multiplex fluorescence immunohistochemistry shows the expression of CD68, CD11B, CD3, and CD31 in different GBOs.

Whole exome sequencing was performed on three randomly selected GBO samples and their corresponding parental tumor tissues to determine whether GBOs retained the genomic mutations in the parental tumor tissues. Based on the somatic variations listed in the GBM genomics study, the GBOs exhibited somatic variations similar to those in the parental tumor tissues (**Figure** **3**A). These three paired tissues also displayed heterogeneity in the type point mutations (Figure 3B). Consistency in the number of shared and private mutations, including single nucleotide variants, was observed (Figure 3C). Similar copy number variations (CNVs) were detected in GBOs and parental tumor tissues (Figure S4, Supporting Information).

### 2.5 The scRNA‐seq Reveals That Cultivated GBOs Mimic the TME

We selected three GBO samples along with their corresponding original tumor tissues for single‐cell transcriptome sequencing analysis, aiming to explore the cell types within GBOs (Figure 3D). Our results indicate that, overall, GBOs and GBMs possess consistent cell populations, both being composed of 21 distinct cell clusters. Moreover, GBO samples have a similar proportion of cell clusters to their corresponding GBMs (Figure 3E). After differentiating between normal cells and malignant tumor cells, we identified eight subsets including astrocytes, endothelial cells, microglia, and T cells (Figure 3E,F). All three pairs of samples exhibit similarities in the subset distribution (Figure 3F), which further validates the consistency between GBOs and the original tumor tissues. Based on this, we further conducted a subset analysis of tumor‐associated macrophages in GBOs. The results show that tumor‐associated macrophages have diverse subsets (Figure 3G), and their distribution is consistent with that of tumor‐associated macrophages in the original GBMs. Subsequently, by using multiplex fluorescence immunohistochemistry to stain the immune cells in different GBO samples, we also found that there are abundant CD68+/CD11B+ cells in GBOs, while CD3+ cells are scarce, and CD31+ cells are present (Figure 3H). These findings suggest that there are immune cells within GBOs, thus mimicking certain characteristics of the tumor microenvironment.

On this basis, one GBO sample was randomly selected again for scRNA‐seq. This GBO was mainly composed of nine different cell clusters (Figure S5A,F, Supporting Information). Subsequent sub‐grouping identified astrocytes, tumor‐associated macrophages, T cells, etc. (Figure S5A,G, Supporting Information). Among them, the number of tumor‐associated macrophages of the M2 type was significantly larger than that of the M1 type (Figure S5A, Supporting Information). Immunohistochemical staining for CD206 and CD3 confirmed the presence of M2‐type macrophages and T cells in the GBO (Figure S5E, Supporting Information). Dimensionality reduction clustering was performed again based on cell‐cycle and differentiation related genes, and the tumor cells were divided into seven subgroups (Figure S5B, Supporting Information). Further analysis of the communication between tumor cells and non‐tumor cells showed that the interaction between M2‐type and M1‐type macrophages was the strongest (Figure S5C,D,H,I, Supporting Information). In addition, extensive interactions were also observed between the subgroups of M2‐type and M1‐type macrophages and the rapidly proliferating tumor cell subgroups. This finding emphasized the dominant role of macrophages in the construction of the GBM tumor microenvironment (Figure S5C,D, Supporting Information).

In summary, the scRNA‐seq analysis indicated that GBOs possess an immune microenvironment primarily composed of M2‐type macrophages that mimic the original TME. These findings supported using GBOs as optimal substitutes for corresponding tumor tissues.

### 2.6 Optimized Culturing Yields Abundant γδ T Cells with High Purity

**Figure 4:**
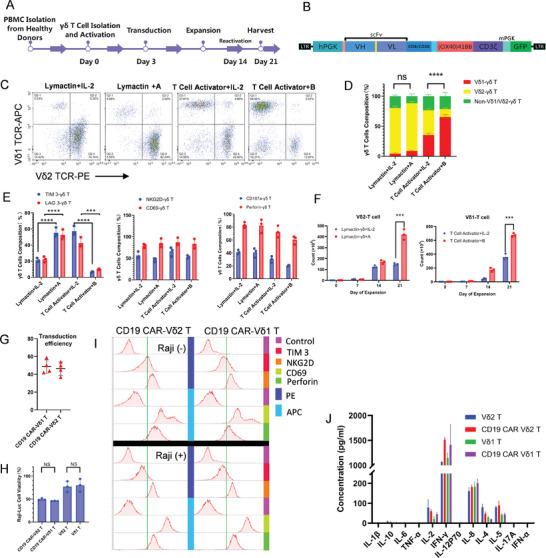
Optimized γδ T cell expansion protocol for selective cultivation of Vδ1 or Vδ2 subtypes. A) Flowchart of γδ T cell expansion. B) CAR structure schematic. C,D) Proportions of γδ T cell subtypes from PBMCs after optimized γδ T cell expansion (*n* = 3). ***P < 0.001 using one‐way analysis of variance with Tukey's post‐hoc test. More detailed data are presented in Figure S6A, Supporting information. E) Proportions of exhaustion and cytotoxic phenotypes of γδ T cells in each group (*n* = 3). ***P < 0.001, ****P < 0.0001 using one‐way analysis of variance with Tukey's post‐hoc test. F) Optimized γδ T cell expansion protocol shows several hundredfold expansions (*n* = 3). ***P < 0.001 using Student's *t*‐test. G) γδ T cells transduced with CD19 CAR lentivirus have a high transduction efficiency (*n* = 4). H) Bioluminescence detection 12 h after co‐culturing CD19 CAR‐γδ T cells with Raji‐luc cells shows a significant cytotoxic effect (*n* = 3). NS, *P* > 0.05 using one‐way analysis of variance with Tukey's post‐hoc test. I) CD19 CAR‐γδ T cells show low exhaustion and high cytotoxic phenotypes. J) CD19 CAR‐γδ T cells do not produce excessive cytokines during the killing of tumor cells. All data are presented as the mean ± SD of triplicate wells.

The culture protocol was optimized using previous γδ T cell culture protocols and incorporating the latest concepts and methods^[^ ^34^, ^35^, ^36^ ^]^ (**Figure** **4**A). The γδ T cells with dominant subsets (the proportion was >60%) were selected. A final culture predominantly consisting of Vδ1 T cells (the proportion was ≈70%) was obtained using a combination of T cell activators and cytokine mix B. These results are in contrast to the <40% purity obtained in the single cytokine group (Figure 4C,D, and Figure S6A, Supporting Information). Phenotypic analysis of Vδ1 T cells cultured under this protocol revealed a highly cytotoxic phenotype with low exhaustion (Figure 4E and Figure S6B, Supporting Information). Vδ1 T cells expanded over 600‐fold in a 21‐d culture period (Figure 4F). A previous strategy was used because of the dual nature of Vδ1 T cells (Vδ1 T cells expressing high FITC anti‐human amphiregulin (AREG) levels promote tumor proliferation), which included secondary stimulation and induction with IL‐15/IL‐18 to achieve similar results. The cultured Vδ1 T cells also exhibited minimal AREG expression (Figure S6C, Supporting Information), thereby enhancing its utility.

γδ T cells, predominantly composed of Vδ2 T cells, were cultivated using the Lymactin antibody for activation and cytokine mix A for induction (Figure 4C and Figure S6A, Supporting Information). The group treated with cytokine mix A for sustained proliferation achieved higher cell quantities than the single IL‐2 group (Figure 4F). However, the single IL‐2 group maintained a similar Vδ2 T cell purity and cytotoxic phenotype to the cytokine mix A group. In addition, the IL‐2 group exhibited lower exhaustion than the cytokine mix A group, possibly due to overstimulating Vδ2 T cells with multiple cytokines (Figure 4E and Figure S6B, Supporting Information).

### 2.7 Engineered CAR‐γδ T Cells Display High Proliferative Potential, Cytotoxicity

γδ T cell transduction was evaluated using an anti‐CD19 CAR lentivirus to assess the applicability of the culture protocol to immunotherapies that require genetic modification (Figure 4A,B). Consistent transduction efficiency (32.01%–58.64%) was observed across eight γδ T cell groups cultured from four different donors (activated with T cell activators or Lymactin) (Figure 4G and Figure S7A, Supporting Information). The number of CD19 CAR‐γδ T cells was quantified to assess the impact of CAR viral transduction on γδ T cell proliferation. Vδ1 T and Vδ2 T cells both exhibited >300‐fold expansion at the 21‐d harvest (Figure S7B, Supporting Information).

CAR‐γδ T cells were co‐cultured with CD19^+^ Raji cells (Figure S7C, Supporting Information) at a 1:1 effector‐to‐target ratio for 7 d for continuous proliferation assessment. CAR‐γδ T cells were highly activated under CD19 antigen stimulation, displaying sustained proliferation. Comparative CAR‐Vδ1 and CAR‐Vδ2 T cell analysis revealed relatively consistent proliferation rates (Figure S7D, Supporting Information), which laid the foundation for subsequent treatments.

Subsequently, the cytotoxic performance of CD19 CAR‐γδ T cells was evaluated using CD19^+^ hematological tumor Raji‐luc cells. CD19 CAR‐γδ T cells exhibited highly efficient killing of CD19^+^ Raji cells compared to non‐transduced CAR‐γδ T cells when combining flow cytometry and bioluminescence imaging (Figure 4H and Figure S7E,F, Supporting Information).

Flow cytometry analysis of phenotypic changes in CD19 CAR‐γδ T cells pre‐ and post‐killing revealed that the TIM‐3 phenotype in γδ T cells remained low, whereas cytotoxic phenotypes such as CD69, NKG2D, and perforin maintained high expression levels. Peak levels shifted toward higher CD69, NKG2D, and perforin levels (Figure 4I). Analyzing multiple cytokines in the supernatant collected after co‐culture demonstrated that CAR‐γδ T cells primarily produced interferon (IFN)‐γ, with minimal amounts of IL‐2, IL‐18, IL‐17, and IL‐5 (Figure 4J). The minimal release of cytokines during the killing process by CAR‐γδ T cells may mitigate the risk of cytokine storms.

### 2.8 CAR‐γδ T Cell Cocktail Eradicates LN229 Spheroids

**Figure 5:**
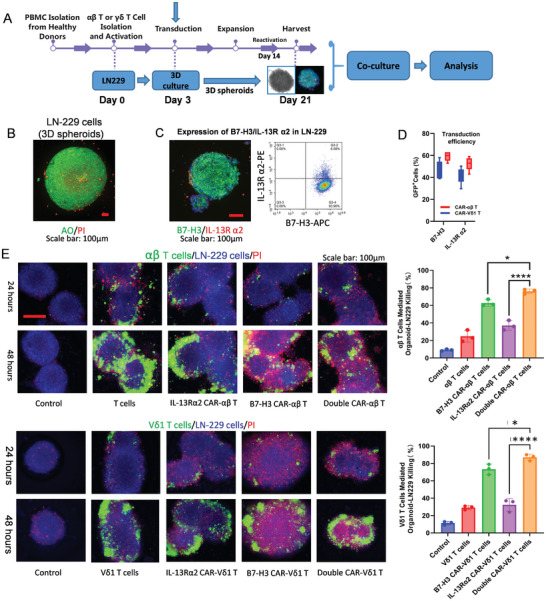
Double CAR‐γδ T cells significantly enhance the killing effect on LN229 spheroids. A) Preparation of double CAR‐γδ T cells and simultaneous corresponding LN229 spheroid preparation. B) Mature LN229 spheroids AO/PI staining. Scale bar: 100 µm. C) Immunofluorescence staining and flow cytometric analysis to detect B7‐H3 and IL‐13Rα2 expression in LN229 cells. Scale bar: 100 µm. D) Transduction efficiency of double CAR‐αβ and CAR‐γδ T cells (*n* = 3). E) Double CAR‐γδ T cells significantly enhance the killing effect (the proportion of PI‐positive cells) on LN229 spheroids (*n* = 3). Scale bar: 100 µm. *P < 0.05, ****P < 0.0001 using one‐way analysis of variance with Tukey's post‐hoc test.

Spheroids were rapidly generated in 3D cultures using the GBM cell line as a foundation to obtain physiologically relevant data. LN229 cells were subjected to 3D culture, resulting in mature LN229 spheroids that were ethically non‐impacting and amenable to repeated sampling (**Figure** **5**A,B). These spheroids exhibited good roundness, robust growth, and sustained cultivation (>2 months). AO/PI staining confirmed that spheroid viability exceeded 95% (Figure 5B). Retrieval from the Cancer Cell Line Encyclopedia (CCLE) database indicated the heterogeneous expression of target genes in common GBM cell lines (Figure S8A, Supporting Information). LN229 cells, in particular, exhibited high B7‐H3 and low IL‐13Rα2 expression. Immunofluorescence staining of LN229 spheroids (B7‐H3, IL‐13Rα2) revealed predominantly B7‐H3‐positive cells, with a small subset displaying IL‐13Rα2 positivity (Figure 5C). B7‐H3 and IL‐13Rα2‐positive LN229 cells constituted almost 100% of the cell population (Figure 5C and Figure S8B, Supporting Information). These findings indicated that LN299 spheroids partially emulate tumor heterogeneity in vivo.

Because of the homing characteristics of these cells, CAR‐Vδ1 T cells which are more suited for solid tumor destruction were prepared following the process outlined in Figure 5A. CAR‐αβ T cells were prepared as a control. The transduction efficiency of B7‐H3 or IL‐13Rα2 CAR was >40% (Figure 5D and Figure S8C, Supporting Information). After the completion of multiple CAR‐γδ T cell preparations, co‐culturing with LN229 spheroids at a 1:1 effector‐to‐target ratio revealed that double CAR‐Vδ1 T cells—targeting B7‐H3 and IL‐13Rα2 jointly—exhibited a similar killing efficiency on LN229 spheroids as that of double CAR‐αβ T cells. The combination therapy demonstrated a more effective killing rate than single target therapy even though most LN229 cells were B7‐H3 positive before the heterogeneity assessment. Non‐transduced Vδ1 T cells or αβ T cells showed limited killing efficiency (Figure 5E).

### 2.9 γδ T Cells Can Infiltrate and Proliferate in GBOs

**Figure 6:**
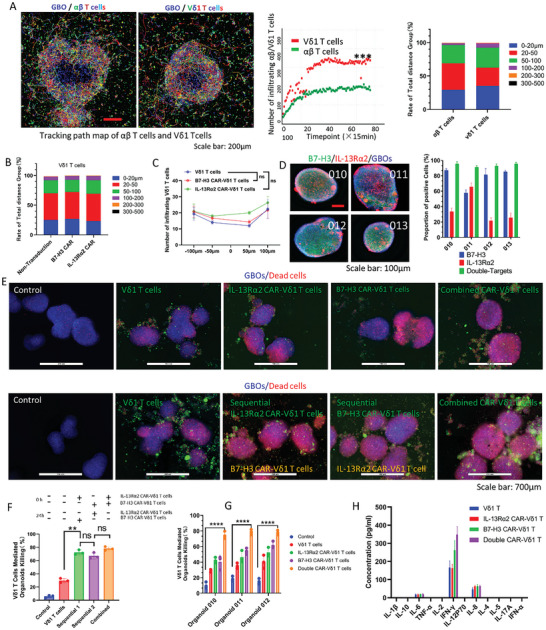
Infiltration characteristics of γδ T cells and therapeutic efficacy of multiple (double) CAR‐γδ T cell cocktail immunotherapy in GBOs. A) Vδ1 T cells have a higher infiltration level than αβ T cells. Scale bar: 200 µm. ***P < 0.001 using the Student's *t*‐test. B) Vδ1 T cells were grouped based on their movement distances, and the proportions of each group were analyzed. C) Infiltration analysis demonstrated a roughly similar infiltration pattern in GBOs for double CAR‐Vδ1 T cells (*n* = 3). NS, *P* > 0.05 using one‐way analysis of variance with Tukey's post‐hoc test. D) Heterogeneous expression and quantification of B7‐H3 and IL‐13Rα2 in different GBOs. Scale bar: 100 µm. E,F) Efficacy and quantification of double CAR‐γδ T cell immunotherapy on GBOs (*n* = 3). Scale bar: 700 µm. **P < 0.01, NS, *P* > 0.05 using one‐way analysis of variance with Tukey's post‐hoc test. G) Quantification of double CAR‐γδ T cell immunotherapy exhibits killing effects on GBOs from different tumor sources (*n* = 3). ****P < 0.0001 using one‐way analysis of variance with Tukey's post‐hoc test. H) After CAR‐Vδ1 T cells mediate the killing of GBOs, cytokines mainly consisting of IFN‐γ are released.

The infiltrative nature of Vδ1 T cells in GBOs was investigated because of the natural tissue infiltration and residency properties of the Vδ1 T cells. Vδ1 T cells exhibited higher infiltration levels and longer movement distances than conventional αβ T cells (**Figure** **6**A and Movie S1, Supporting Information). A path map tracking Vδ1 T cells revealed that two Vδ1 T cell groups transduced with different CAR genes exhibited infiltration consistent with that of non‐transduced Vδ1 T cells (Figure S9A, Supporting Information). Further distance grouping analysis of Vδ1 T cell movement trajectories demonstrated their consistency (Figure 6B). Vδ1 T cell infiltration in different GBOs was analyzed, which showed similarity (Figure S10A, Supporting Information). The infiltrative nature of CAR‐Vδ1 T cells was analyzed using the region‐based method (Figure S10C, Supporting Information). B7‐H3 CAR‐Vδ1 and IL‐13Rα2 CAR‐Vδ1 T cells consistently exhibited similar infiltration patterns in different GBO samples (Figure 6C and Figure S10B, Supporting Information), thereby assuring the implementation of subsequent multiple CAR‐γδ T cell cocktail immunotherapy.

### 2.10 Multiple CAR‐γδ T Cell Cocktail Immunotherapy Exhibits Enhanced Therapeutic Efficacy

B7‐H3 and IL‐13Rα2 expression in GBOs was determined using immunofluorescence staining. Heterogeneous expression of target antigens in both the intra‐ and inter‐tumor regions was observed, emphasizing the antigenic diversity of the tumor (Figure 6D and Figure S11A, Supporting Information). Overlaying B7‐H3‐positive cells with IL‐13Rα2‐positive cells yielded a proportion exceeding 90% (Figure 6D). B7‐H3, IL‐13Rα2 CAR‐Vδ1, or both T cells were used to assess cytotoxicity in three distinct GBOs to validate the effectiveness of double CAR‐γδ T cell immunotherapy. Significantly enhanced cytotoxic effects were observed in the combination therapy group, with most tumor cells eradicated within 48 h (Figure 6E–G and Figure S11B, Supporting Information). Remarkably, sequential treatment with double CAR‐γδ T cells exhibited efficacy comparable to that of combined therapy (Figure 6E, F).

γδ T cells co‐cultured with GBOs for 48 h were collected, and flow cytometry was conducted to examine the phenotypic changes in γδ T cells after GBO cytotoxicity. A low‐exhaustion (LAG3, PD‐1 positive) and high‐level cytotoxic phenotype (CD69, NKG2D, CD107a) were observed across all γδ T cell groups (Figure S11C, Supporting Information). γδ T cells consistently maintained low cytokine levels, with only a mild increase in IFN‐γ release. The release of other cytokines was minimal or negligible, notably lacking IL‐17 release (Figure 6H).

Furthermore, GBOs co‐cultured with γδ T cells for 48 h were collected to assess the specificity of double CAR‐γδ T cells against B7‐H3 or IL‐13Rα2‐positive cells in GBOs. A significant reduction in fluorescence intensity of B7‐H3 or IL‐13Rα2‐positive cells was observed in the combined therapy group, confirming the specificity of double CAR‐γδ T cell immunotherapy and its pronounced improved efficacy (Figure S9B,C). The results of flow cytometry analysis showed that the proportion of CD163‐positive cells among CD68‐positive cells decreased significantly after the infiltration of B7‐H3 CAR‐Vδ1 T cells into GBOs (Figure S9D,E, Supporting Information).

**Figure 7:**
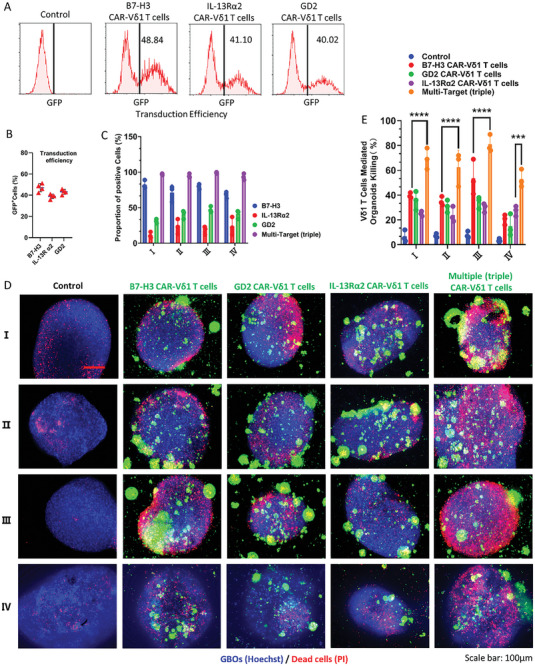
Cytotoxic effects of multiple (triple) CAR‐Vδ1 T cell cocktail on GBOs (I, II, III, and IV represent tumor samples from different patients.). A,B) Flow cytometry was used to assess the transduction efficiency of B7‐H3, IL‐13Rα2, and GD2 CAR‐γδ T cells. C) Quantitative expression of B7‐H3, IL‐13Rα2, and GD2 target heterogeneity based on immunofluorescence staining. D) Cytotoxic effects of triple CAR‐Vδ1 T cell immunotherapy on GBOs. Scale bar: 100 µm. E) Quantification graph depicting the cytotoxic effects of multiple (triple) CAR‐Vδ1 T cell cocktail therapy on GBOs (*n* = 4). The data are presented as the mean ± SD of triplicate wells. ***P < 0.001, ****P < 0.0001 using one‐way analysis of variance with Tukey's post‐hoc test.

The effectiveness of triple CAR‐γδ T cells was then assessed. Initially, B7‐H3, IL‐13Rα2, and GD2 CAR‐γδ T cells were obtained through a standardized viral transduction protocol, with flow cytometry confirming transduction efficiencies exceeding 40% (**Figure** **7**A,B). B7‐H3, IL‐13Rα2, and GD2 were heterogeneously expressed in different GBOs. The overlay of the triple‐target‐positive cells covered >95% of the entire GBO population (Figure 7C and Figure S12, Supporting Information). Combination therapy involving triple CAR‐γδ T cells exhibited significant cytotoxic effects, with most tumor cells being eradicated within 24 h (Figure 7D,E). These findings underscored the improved therapeutic efficacy of multiple CAR‐γδ T cell cocktail immunotherapy.

### 2.11 Rational Combination of Multiple (Double) CAR‐Vδ1 T Cell Cocktail Therapy Achieves Optimal Anticancer Benefits in Tumor‐Bearing (GBO‐luc) Mice

**Figure 8:**
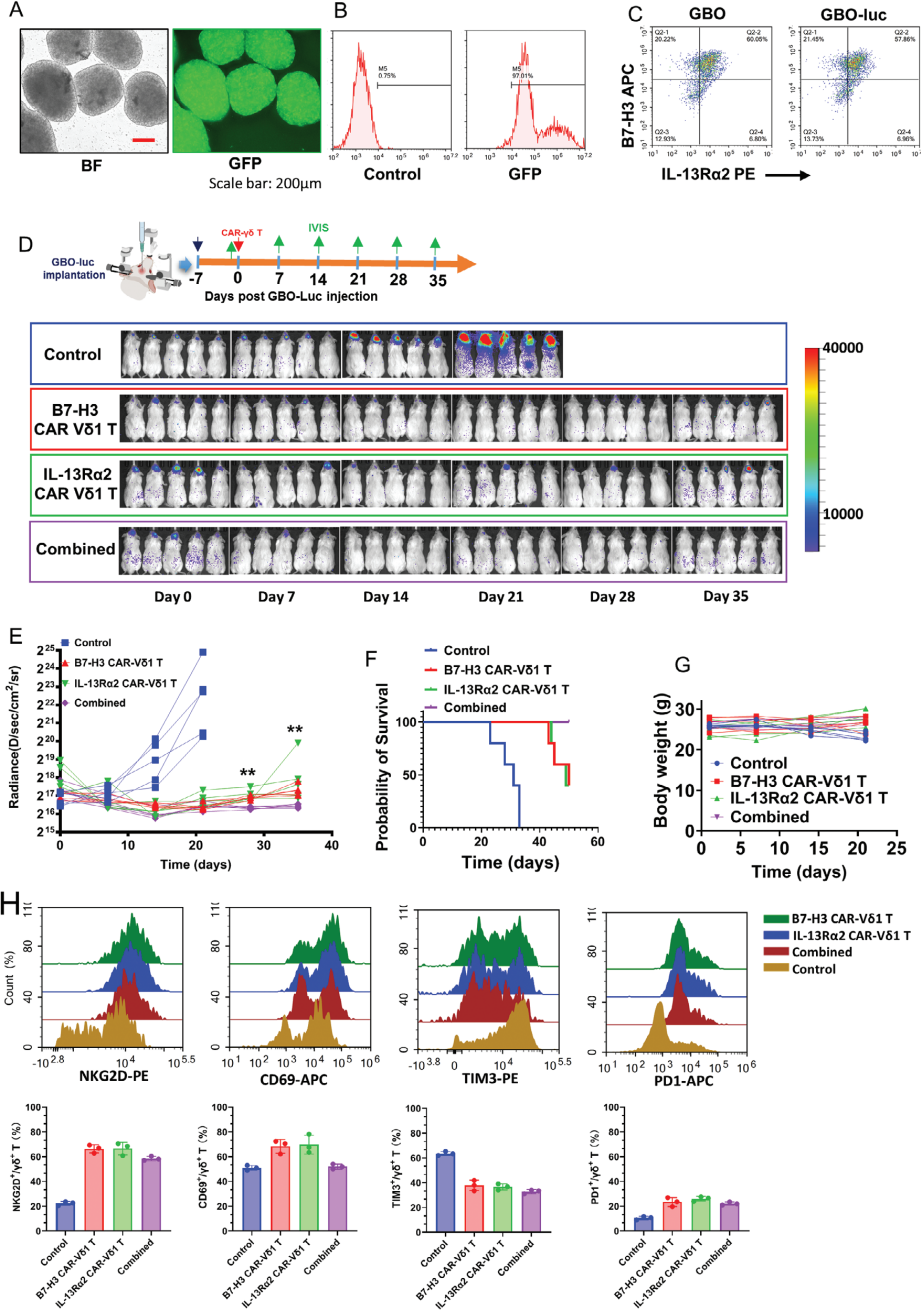
The killing effect of the multiple (double) CAR‐Vδ1 T cell cocktail therapy on the orthotopic xenograft tumor‐bearing mouse model constructed from patient‐derived GBOs. A,B) The GBO‐luc was constructed by transducing GBO with the Luc‐GFP lentiviral vector, followed by fluorescence identification. Scale bar: 200 µm. C) After GBO was transduced by the Luc‐GFP lentiviral vector, the expression levels of both B7‐H3 and IL‐13Rα2 remained stable. D) Flowchart of multiple (double) CAR‐Vδ1 T cell treatment for orthotopic xenograft tumor‐bearing (GBO‐luc) mouse model. D,E) The IVIS was used to evaluate the efficacy and quantify the effects of multiple (double) CAR‐Vδ1 T cells therapy in tumor‐bearing (GBO‐luc) mice. *n* = 5/each group. F,G) Survival analysis of orthotopic xenograft tumor‐bearing mice treated with multiple CAR‐Vδ1 T cells and the changes in body weight during the treatment period. H) Phenotypic analysis of CAR‐γδ T cells infiltrating into tumor tissues of different groups.

After confirming the expression of the double targets in GBO‐luc (**Figure** **8**A–C), following the procedure outlined in Figure 8D, an orthotopic xenograft mouse model of GBO‐luc was constructed. The In Vivo Imaging System spectrum (IVIS Spectrum) demonstrated that the model was successfully established, and then stereotactic injection of an equal amount of multiple CAR‐Vδ1 T cells was performed. The IVIS Spectrum revealed that the treatment group with double CAR‐Vδ1 T cells exhibited a faster tumor regression effect, significantly higher than the single B7‐H3 or IL‐13Rα2 CAR‐Vδ1 T cell group (Figure 8D,E). During the limited observation period, the mouse models in the double CAR‐Vδ1 T cell treatment group even showed persistent anti‐tumor ability, with most of the tumor cells cleared, while the single target treatment group inevitably experienced tumor recurrence. Survival analysis also indicated a significant extension of the survival period of mouse GBM models in the double CAR‐Vδ1 T cell treatment group (Figure 8F). Throughout the entire observation process, no signs of GvHD were observed in any group of mice GBM models. The body weights of the mice in the treatment group remained stable (Figure 8G). At the treatment endpoint, the mice were euthanized, and tissues from the liver, kidney, brain, and lungs were harvested after perfusion with formaldehyde. Standard H&E staining revealed no immunotherapy‐induced organ damage in mouse tissues, including the heart, liver, spleen, lung, and kidney (Figure S13, Supporting Information). By performing phenotypic analysis on CAR‐γδ T cells infiltrating into tumor tissues after treatment in different groups, we found that the CAR‐γδ T cells infiltrating into tumor tissues still maintained a high‐level killing phenotype and a low‐level exhaustion phenotype (Figure 8H).

## 3 Discussion

In this study, we developed a multiple CAR‐γδ T cell cocktail therapy specifically addressing the heterogeneity of GBM. This personalized immunotherapy approach is designed to counteract tumor immune resistance caused by tumor heterogeneity during specific‐targeting immune cell therapy. We first screened a set of antigens heterogeneously expressed in GBM using literature review and bioinformatics analysis and formed a target‐bank. Personalized panels of antigens were then selected for each patient based on multiplex immunohistochemistry results, ensuring broad coverage of cancer cells. Using Vδ1 T cells (with natural infiltration) as CAR vehicles, we optimized their engineering for high purity and cytotoxicity, and tested these CAR‐Vδ1 T cell cocktails in patient‐derived GBM organoids, demonstrating superior efficacy compared to single‐targeting CAR‐Vδ1 T cells. Our work highlights the feasibility and potential impact of the “***prof***” multi‐targeted strategy, demonstrating its ability to overcome the limitations of fixed‐menu immunotherapies and advance personalized cancer treatment.

Unlike hematologic malignancies, the heterogeneity of solid tumors such as GBM often results in less effective single CAR‐T cell therapies. CAR T‐cell therapy faces obstacles in solid tumors, such as tumor heterogeneity, antigen loss, and an immunosuppressive microenvironment.^[^ ^37^ ^]^ Tumor heterogeneity particularly complicates CAR‐T cell targeting, and multi‐target antigen combination therapy is an effort to overcome these challenges.^[^ ^38^ ^]^ Understanding the types and spatial distribution of target antigens within and between GBM tumors presents a challenge for designing personalized, nearly comprehensive multiple CAR‐T cell therapies. Fortunately, advancements in neurosurgical and stereotactic techniques have made it relatively easy and low‐risk to acquire tumor samples from GBM patients, facilitating individualized multi‐antigen screening. In our extensive CAR‐T cell research, we have accumulated a substantial amount of data and information related to targets. The target‐bank^[^ ^39^, ^40^ ^]^ related to GBM includes B7‐H3,^[^ ^41^ ^]^ IL‐13Rα2,^[^ ^42^, ^43^ ^]^ Her2,^[^ ^44^, ^45^ ^]^ GD2,^[^ ^46^, ^47^ ^]^ CD133,^[^ ^48^ ^]^ NKG2DL,^[^ ^49^ ^]^ CD70,^[^ ^50^ ^]^ EphA2,^[^ ^51^ ^]^ and EGFRvIII.^[^ ^52^, ^53^ ^]^ In order to target these antigens, a corresponding CAR‐bank was simultaneously designed (not shown in this paper). On this basis, we combined the expression rates of common GBM targets from the literature with the expression levels of relevant genes in the TCGA database to select B7‐H3, IL‐13Rα2, Her2, and GD2 as the target pool for our multiple CAR‐γδ T cell cocktail therapy at present. We then validated the spatial distribution and heterogeneity of these predefined common targets using multiplex immunofluorescence and panoramic scanning techniques. Through the analysis of different tumor samples, we found that combinations of dual‐target antigens, such as B7‐H3 and IL‐13Rα2, can cover more than 80% of tumor regions. This combination shows high coverage in most samples, even in tumors where both targets are relatively lowly expressed. Further analysis indicates that adding a third target, such as GD2, can increase coverage to over 95%. This combination can function across a broader range of tumor areas, reducing the impact of antigen loss on treatment efficacy. To simplify the program, the combination of four targets will not be calculated temporarily. Therefore, personalized dual or triple CAR‐T cocktail therapies can minimize antigen escape to the greatest extent. Given that the B7‐H3 and IL‐13Rα2 combination shows higher coverage than other combinations across different patient tumor samples, this combination has a certain level of general applicability. It is undoubtedly an excellent choice for patients who cannot provide tumor samples.

We are well aware that tumor heterogeneity is one of the key challenges in the treatment of GBM, and our elaborately designed individualized multiple target strategy is based on a full consideration of this challenge. First, we conducted an in‐depth analysis of the currently available GBM target data and constructed a comprehensive target library. Subsequently, we used mIHC to detect GBM samples, analyzed the expression of some targets in the target library, and then determined the coverage ratio of different combinations of common targets on tumor cells. Through this approach, we ensure that individualized multiple CAR‐γδ T cells can act on tumor cells with different phenotypes to the greatest extent, thus effectively addressing the problem of tumor heterogeneity.

To provide more powerful experimental evidence, based on the verification of in‐vitro tumor organoids, we further carried out in‐vivo experiments of multiple CAR‐γδ T cells using an orthotopic xenograft animal model sourced from heterogeneous GBOs. Specifically, we inoculated the constructed heterogeneous patient‐derived GBO‐luc into the intracranial region of NCG mice, successfully establishing a highly heterogeneous orthotopic tumor model. Subsequently, we treated the mice with multiple CAR‐γδ T cells and used bioluminescence imaging technology to monitor the growth trend of tumors in real‐time. The experimental results show that the tumor growth of the mice in the treatment group was significantly inhibited, while tumor recurrence in the single target CAR‐γδ T cells treatment group was inevitable during the observation period. This fully demonstrates that CAR‐γδ T cells can also efficiently recognize and attack heterogeneous tumor cells in vivo.

Preclinical studies of immune cell drugs require a high degree of tumor model simulation. Humanized immune system mouse tumor models, PDX models, and others face drawbacks such as complex processes, long cycles, and poor simulation fidelity. Even the recently developed tumor organoid models encounter difficulties in constructing the tumor microenvironment. We addressed this challenge by establishing GBO models derived from GBM tissue fragments using a microgravity 3D cell culture system. This non‐single‐cell dissociation method uses a specific culture medium devoid of exogenous growth factors or extracellular matrix, which better preserves intra‐ and intertumoral heterogeneity and retains the essential features of the parental tumor compared to single‐cell dissociation methods.^[^ ^33^ ^]^ Histological, molecular, and genomic analyses confirmed that the GBOs maintained key characteristics of the original tumors, including both intra‐ and intertumoral heterogeneity. Notably, the GBOs retained the predominant population of immunosuppressive M2‐type macrophages found in the original tumor tissue. Further analysis of the communication between macrophages and tumor cells within the GBOs revealed robust cell‐cell interactions, particularly between M1 and M2 macrophages, supporting the hypothesis that GBOs maintain the functional aspects of the original tumor tissue, thereby simulating the immunosuppressive TME. Most GBOs can be generated within 5–8 d under standard sampling and timely culture conditions, which has substantial implications for the individualized treatment of patients with GBM.

As a crucial component of the “***prof***” strategy, appropriate engineered T cell products are essential for the efficacy of multiple CAR‐T cell cocktail therapy. Compared to αβ T cells, γδ T cells possess natural homing ability, broad‐spectrum antigen recognition, and low cytokine release, making them a promising new tool for immune cell therapy. Specifically, allogeneic universal γδ T cell products from carefully selected healthy donors can be pre‐fabricated into off‐the‐shelf CAR‐γδ T cell products targeting multiple antigens. This significantly reduces the cost of CAR‐T cell therapy and alleviates the financial burden on patients. More importantly, it greatly reduces patient waiting times. Although several expansion protocols for Vδ1 T cells targeting cancer have been developed,^[^ ^35^, ^54^ ^]^ and CAR‐Vδ1 T cells have shown potent anti‐tumor functions in vitro and in vivo, triggering innate and adaptive immune responses,^[^ ^55^ ^]^ large‐scale expansion remains challenging, and controversies about the anti‐tumor effects of tissue‐homing Vδ1 T cells persist.

In this study, we developed an optimized γδ T cell culture method based on various Vδ1 T cell expansion protocols, achieving high‐purity, highly active, and abundant γδ T cells with low cytokine release. By using commercial T cell activators for γδ T cell activation and maintaining the phenotype with a cytokine mixture, we obtained a higher proportion of Vδ1 T cell subsets with effective anti‐tumor functions. Recent studies have shown that AREG protein expression is associated with the pro‐tumor phenotype in Vδ1 T cells,^[^ ^36^ ^]^ and our optimized cultured γδ T cells almost completely lacked AREG protein expression. On the other hand, while extreme purification of Vδ1 T cell subsets can be achieved through magnetic selection, research indicates that extreme purity does not necessarily correspond to superior clinical efficacy. Maintaining TCR diversity or an appropriate γδ T cell subset ratio is advantageous for antigen recognition.^[^ ^35^ ^]^ However, further research is needed to determine the optimal ratio of these populations. On the one hand, it is necessary for us to first clarify the efficacy, mechanism of action, and potential problems of the CAR‐γδ T cell therapy when applied alone, providing solid basic data and theoretical support for the design and optimization of subsequent combination therapies. On the other hand, combination immunotherapy involves numerous complex factors, such as the interactions between different immune cells or drugs, dose optimization, administration sequence, and potential superposition of toxic and side effects.^[^ ^56^, ^57^, ^58^ ^]^ All these require more systematic and in‐depth preliminary research. If combination therapy is introduced too early, then it may be difficult to accurately analyze the core value and mechanism of action of the CAR‐γδ T cell therapy itself due to the interference of multiple factors, which is not conducive to the gradual in‐depth and precise progress of the research.

Next, to simulate the physiological relevance and intra‐tumoral heterogeneity of solid tumors, this study used 3D‐cultured LN229 spheroids as an initial model. This 3D‐cultured model provided a physiologically relevant solid tumor foundation for testing the multiple CAR‐Vδ1 T cell cocktail therapy. Our results indicate that the multiple CAR‐Vδ1 T cell cocktail therapy exhibits superior tumor cell clearance compared to CAR‐αβ T cells in the same batch. As a solid tumor 3D model derived from a cell line, LN229 spheroids are excellent; however, they can only partially simulate tumor heterogeneity and lack the support of the tumor microenvironment, limiting their application. Therefore, patient‐derived GBO models are undoubtedly the closest to the real state and provide a more accurate representation for further testing and validation.

The difficulty of infiltrating solid tumors is a major reason for the poor efficacy of immune cell therapy for malignant tumors. However, Vδ1 T cells with natural tissue‐homing abilities can infiltrate solid tumors and kill tumor cells. Traditional tumor models often lack a realistic immune microenvironment, limiting the accuracy of research results. GBO models, which incorporate immune microenvironments, offer significant advantages in simulating tumor microenvironments, providing a more realistic foundation for evaluating cell infiltration and therapeutic efficacy. To evaluate the infiltrative capacity of Vδ1 T cells, we used patient‐derived GBO models that mimic the TME. Experimental results showed that Vδ1 T cells demonstrated stronger infiltration capabilities compared to αβ T cells. Furthermore, Vδ1 T cells infiltrating GBOs exhibited rapid proliferation and a broader range of activity. Region‐based infiltration analysis revealed that Vδ1 T cells transduced with various CARs could infiltrate the entire GBO, including central locations. These findings provide essential assurance for maintaining therapeutic efficacy in solid tumors. Multiple CAR‐γδ T cell cocktail therapy demonstrates excellent tumor‐killing effects regardless of whether dual‐ or triple‐target strategies were used. This therapy maintains a high level of killing capacity and a low level of cell exhaustion phenotype. Multiple CAR‐γδ T cells only release IFN‐γ, which has an auxiliary killing effect, thereby avoiding the cytokine storm complications commonly seen in CAR‐αβ T cell treatments. Notably, research indicates that the immunosuppressive TME can polarize cytotoxic γδ T cells into IL‐17‐producing immunosuppressive cells,^[^ ^59^ ^]^ yet in our study, the Vδ1 T cells used did not release IL‐17, emphasizing the important role of multiple CAR‐γδ T cells in maintaining continuous tumor‐killing ability. Based on these findings, the “***prof”*** strategy can comprehensively and accurately evaluate patient responses to immunotherapy and provide personalized multiple CAR‐γδ T cell cocktail therapy.

In the tumor microenvironment, the interactions among immune cells are indeed extremely complex and crucial. Especially in the treatment of GBM, regulating the tumor microenvironment to enhance the immune response is of vital importance.^[^ ^60^ ^]^ In our study, we also conducted a preliminary exploration of the close interactions and potential regulatory mechanisms between the CAR‐γδ T cells therapy and various immune cells in the tumor microenvironment. First, tumor‐associated macrophages play a significant role in the GBM microenvironment. Research shows that they exist in two polarization states: the M1 type (with anti‐tumor effects) and the M2 type (with pro‐tumor effects). Tumor cells can prompt macrophages to polarize towards the M2 type. M2‐type macrophages secrete a variety of immunosuppressive factors, which inhibit the anti‐tumor activities of T cells, NK cells, etc., creating a microenvironment conducive to tumor growth.^[^ ^61^ ^]^ IFN‐γ can reverse the polarization state of macrophages, transforming them from the M2 type to the M1 type and enhancing the local anti‐tumor immune response.^[^ ^62^ ^]^ The cytokines secreted by γδ T cells are mainly IFN‐γ. We analyzed the macrophage population after co‐culturing GBO, which can simulate the microenvironment, with CAR‐γδ T cells. The results showed that after treatment with CAR‐γδ T cells, the proportion of M2‐type macrophages in the tumor microenvironment decreased. At the same time, cytokines mainly consisting of IFN‐γ were detected in the co‐culture system, while the amount of immunosuppressive cytokines was minimal. All these provide evidence for the regulatory function of CAR‐γδ T cells on macrophages. Second, T cells also play a central role in tumor immunity, but their functions are often inhibited in the GBM microenvironment. On one hand, immune checkpoint molecules such as PD‐L1, which are highly expressed on the surface of tumor cells, bind to PD‐1 on the surface of T cells, leading to T‐cell exhaustion.^[^ ^63^ ^]^ On the other hand, regulatory T cells in the tumor microenvironment inhibit the activation and proliferation of effector T cells through mechanisms such as secreting inhibitory cytokines and direct contact.^[^ ^64^ ^]^ Substances such as perforin and granzyme secreted by CAR‐γδ T cells can not only directly kill tumor cells but may also act on regulatory T cells, reducing their inhibitory ability and thus breaking the immunosuppressive balance in the tumor microenvironment.^[^ ^65^ ^]^ CAR‐γδ T cells can also function as antigen‐presenting cells to activate αβ T cells. Meanwhile, CAR‐γδ T cells promote the maturation of DCs, enhancing the antigen‐presentation and activation of αβ T cells by DCs.^[^ ^21^ ^]^ At the same time, in return, DCs can induce the activation and proliferation of CAR‐γδ T cells, enhancing their cytotoxicity and immunomodulatory functions.^[^ ^66^ ^]^ CAR‐γδ T cells can induce NK cell mediated anti‐tumor cytotoxicity through the action of CD137.^[^ ^67^ ^]^

Given the current research results, although we have explored the complex relationships between CAR‐γδ T cells and common immune cells in the immune microenvironment, there are still a large number of details and underlying mechanisms that urgently need to be further explored. A comprehensive and in‐depth analysis of these interactions and the basic immunological mechanisms behind them is the cornerstone for formulating effective clinical treatment plans for GBM patients.

According to the current experimental results, we have carefully designed a comprehensive set of clinical trial protocols by referring to the latest clinical trial literature.^[^ ^42^, ^68^, ^69^ ^]^ Our aim is to ensure that this innovative therapy is effective and feasible in clinical practice. First, we have emphasized the crucial significance of individualized target combination screening. This step will accurately determine the most suitable treatment targets according to the unique tumor antigen expression profiles of each patient, thus achieving the goal of individualized precision medicine. Before patients are enrolled, their tumor samples are obtained first. Then, mIHC is used to screen the expression of targets (B7‐H3, IL‐13Rα2, Her2, GD2, CD133, NKG2DL, CD70, and EGFRvIII). According to the expression of these targets, an individualized target combination plan is formulated for each patient. Given the “Off‐the‐Shelf and Universality” characteristics of CAR‐Vδ1 T cells, we have used the peripheral blood of immunocompetent donors to pre‐prepare a series of target CAR‐Vδ1 T cells (CAR‐BANK) that meet clinical standards and are active. This eliminates the need for patients’ autologous blood and the complex T‐cell expansion process. This approach effectively overcomes the potential failure of autologous blood T‐cell expansion in immunocompromised patients, and the Off‐the‐Shelf CAR‐Vδ1 T cells also significantly reduce the waiting time for patients. After obtaining the individualized target combination of patients, we can conveniently resuscitate the corresponding target CAR‐Vδ1 T cells when needed. Secondly, in terms of specific implementation, we have optimized the process. After patients are enrolled, according to their specific conditions, we establish channels for subsequent local drug delivery by implanting an intracranial Ommaya reservoir or combining surgical resection of part of the tumor. At the same time, key parameters such as the time, method, and dose of local injection have been strictly optimized and standardized. In addition, the tumor samples resected surgically can be used to construct tumor organoids to pre‐validate the selected target combination, thereby further improving the success rate and safety of treatment. Although this step is not essential, for some complex cases or patients with the necessary conditions, it can provide additional assurance. Furthermore, to comprehensively monitor the treatment effect and the overall condition of patients, we have established a comprehensive and systematic system of clinical monitoring indicators. This system covers multiple aspects: Hematological indicators are used to detect patients’ immune responses, inflammatory states, and possible cytokine storms; Imaging examinations, such as regular CT and MRI, are used to visually observe the size, shape, and position changes of tumors, so as to evaluate the inhibitory effect of treatment on tumors; Neurological function assessment, considering the potential impact of GBM on the nervous system, we will closely monitor patients’ cognitive, motor, sensory, and other functional indicators to promptly detect and address possible neurotoxic reactions; And quality‐of‐life surveys, which assess patients from multiple dimensions such as their daily living self‐care ability, psychological state, and social activities, ensuring that the treatment not only prolongs patients’ survival but also improves their quality of life (Figure S14, Supporting Information, step 2). At present, we have smoothly passed the ethical review of this clinical protocol and successfully completed the registration on the China Medical Research Registration and Filing Information System and the ClinicalTrials platform (ID: NCT06572956). As of now, we have successfully recruited several patients. The personalized target combination design and the implantation of the Ommaya reservoir have been successfully completed for all of them, and the subsequent work is being steadily carried out in strict accordance with the predetermined plan.

Despite acquiring panoramic data for the entire tumor tissue slice to the maximum extent possible, this study had limitations owing to the 2D nature of the tumor tissue slices and intra‐tumoral heterogeneity. CAR‐γδ T cell products generated from different donors vary. Tumor organoids mimicking the TME encounter challenges, including a decline in immune cell proportions, difficulties in maintaining target antigens, and different cell type migration. Future analyses involving multi‐site sampling and multi‐layer slicing to form spatial big data and subsequent analyses can achieve a more accurate acquisition of target information from tumor samples than single tumor tissue slice. However, continuously expanding a readily available universal CAR‐γδ T cell product library can reduce differences between donors. Prompt analysis of GBOs after establishment helps mitigate the limitation of cell migration. Although γδ T cell immunotherapy holds promise, further research is warranted to better understand the broader immune system functions of γδ T cells in anti‐tumor actions and responses to treatment within heavily immunosuppressive, hypoxic, and metabolically competitive TMEs.^[^ ^70^, ^71^ ^]^ The underlying mechanisms are multi‐dimensional and complex. One essential mechanism of treatment resistance in CAR‐γδ T cell therapy could be attributed to the insufficient persistence and functionality of these cells in the tumor microenvironment. This is a common issue observed in other CAR‐T cell therapies as well, where inadequate persistence and subsequent loss of infused CAR‐T cells are major resistance mechanisms associated with treatment failure.^[^ ^72^ ^]^ In addition, the tumor microenvironment can play a crucial role in mediating resistance. The purpose of our construction of tumor organoids that can simulate the tumor microenvironment is to provide a more realistic and reliable model for evaluating the therapeutic effect of the individualized multiple CAR‐γδ T cells. In GBM, the tumor microenvironment contains various cell components such as tumor‐associated macrophages, regulatory T cells, and fibroblasts, which jointly create an immunosuppressive environment. The immunosuppressive tumor microenvironment can impair the function of CAR‐T cells, including CAR‐γδ T cells. This environment can lead to CAR‐T cell exhaustion, reducing their efficacy over time.^[^ ^73^ ^]^ Furthermore, the density of target antigens on tumor cells can influence the effectiveness of CAR‐T cells. In cases where antigen density is low, CAR‐T cells may not be able to effectively engage and eliminate tumor cells, leading to resistance.^[^ ^74^ ^]^ Moreover, the genetic and phenotypic characteristics of the tumor itself can contribute to resistance. For example, mutations or alterations in target antigens can lead to antigen loss or modification, rendering CAR‐T cells ineffective. This is precisely the original intention and cornerstone of our conception and design of the individualized multiple CAR‐γδ T cell therapy. Admittedly, even with the individualized multiple target strategy, it remains difficult to comprehensively cover all phenotypic tumor cell subsets. This has been observed in other CAR‐T therapies, such as those targeting CD19, where antigen loss is a frequent cause of resistance.^[^ ^75^ ^]^ Understanding these mechanisms is crucial for developing strategies to overcome resistance and enhance the efficacy of CAR‐γδ T cell therapy.

The focus should also extend to reducing polarization effects and potentially reversing immunosuppressive actions in these challenging environments. The heterogeneity of solid tumors poses challenges for CAR‐T cell immunotherapy, and this study has enhanced the efficacy against brain GBM through “***prof***” multiple CAR‐γδ T cell cocktail therapy. GBOs simulating the TME provide a realistic preclinical model for multiple CAR‐γδ T cell cocktail therapy. Importantly, this study introduces an in vitro evaluation strategy for cell therapy that transcends single‐ or fixed‐target combinations. In the future, precise treatments for GBM (and potentially other malignant solid tumors) can be realized through “***prof***” dual‐target, triple‐target, or even more target CAR‐γδ T cell cocktail therapies (Scheme 1 and Figure S14, Supporting Information).

## 4 Experimental Section

### Ethical Approval and Consent to Participate

The Ethics Committee of the Second Hospital of Shandong University approved the study (No. KYLL2024094), and after obtaining the consent of patients, samples of GBM from patients and peripheral blood samples from healthy donors were collected at the Second Hospital of Shandong University. All patient samples have been de‐identified before processing. The use of human GBM samples and peripheral blood samples from healthy donors follows ethical and technical guidelines regarding the use of human samples for biomedical research purposes.

### Cell Lines

The HEK‐293T cell line was procured from the American Type Culture Collection (ATCC, USA) for lentiviral packaging. HEK‐293T cells and human GBM LN229 cells (Servicebio, Wuhan, China) were cultured under standard conditions using Dulbecco's modified Eagle's medium (DMEM) supplemented with 10% fetal bovine serum (GIBCO, USA). The human Burkitt's lymphoma cell line Raji was obtained from ATCC. LN229 and Raji cells expressing luciferase (luc) were generated through lentiviral infection.

### Formation of Target‐Bank and Gene Expression Analysis (Public Databases)

Previous work related to CAR and literature review were accumulated, forming GBM's target‐bank. RNA‐sequencing expression profiles for B7‐H3, Her2, IL‐13Rα2, ST8SIA1, CD70, EGFR, EphA2, CD133, and NKG2DL were downloaded from the TCGA dataset (https://portal.gdc.com). GTEx data from version 8 (https://gtexportal.org/home/datasets) were also incorporated. All the analysis methods and R package were implemented by R version 4.0.3.

### Cell Line Gene Expression Analysis (Cancer Cell Line Encyclopedia Dataset)

The gene expression matrix for GBM cell lines was acquired from the CCLE dataset (https://portals.broadinstitute.org/ccle/about). Differential gene expression across various cell lines was analyzed using heatmap visualization.

### The Public scRNA‐seq Datasets Analysis

The public scRNA‐seq datasets were downloaded from the Gene Expression Omnibus (GEO) database (https://www.ncbi.nlm.nih.gov/geo), specifically GSE131928, GSE138794, and GSE140819, which include data from GBM samples. The Seurat package (version 4.0) was used for data processing. Initially, low‐quality cells were filtered out based on criteria such as removing cells with high mitochondrial gene expression (>20%) or low gene expression (<200 or >2500 genes). The data were then normalized using the “NormalizeData” function to ensure comparability across samples. Highly variable features were identified using the “FindVariableFeatures” function, and the data were centered and scaled using the “ScaleData” function. Principal component analysis (PCA) was performed using the “RunPCA” function to reduce data dimensionality. Malignant GBM cells were identified based on known markers using the “FindMarkers” function and were selected using the “Subset” function for subsequent analysis. Based on literature review and bioinformatics analysis, four genes associated with GBM were selected: B7‐H3 (CD276), IL‐13Rα2 (IL13RA2), Her2 (ERBB2), and GD2 (ST8SIA1). The expression levels of these genes were extracted from the processed datasets and visualized using the “ggplot2” and “Seurat” packages. These visualizations displayed the distribution and co‐expression of the selected genes in GBM samples, revealing the spatial distribution and heterogeneity of the target genes in the tumor microenvironment.

### Multiplex Immunohistochemistry

The mIHC was used to assess the heterogeneity and expression of multiple target antigens in the tumor tissues. Paraffin‐embedded slices were subjected to antigen retrieval, endogenous peroxidase activity blocking, and nonspecific site blocking. Staining was performed using a multilabel immunohistochemical protocol with opal tyramide signal amplification. Primary antibodies included B7‐H3, GD2, Her2, and IL‐13Rα2 rabbit monoclonal antibodies (Cell Signaling Technology, USA), following the manufacturer's instructions. 4′,6‐diamidino‐2‐phenylindole (DAPI) was used for nuclear counterstaining. Images were acquired using the Mantra quantitative pathology imaging analysis system (Perkin‐Elmer, USA), and image processing and analysis were performed using Inform Tissue Finder software v 2.2.1 (Perkin‐Elmer). Representative samples were collected using the TissueFAXS PLUS multispectral panoramic pathology tissue slide scanning system (TissueGnostics, USA), and analysis was conducted using TissueQuest software v 4.0 (TissueGnostics).

### GBO Culture

Freshly resected GBM tissue was pathologically examined by neuropathologists for a preliminary diagnosis of high‐grade glioma. The tissue samples were divided into three parts: GBO culture, whole‐exome sequencing, and paraffin specimen preparation. For GBO culture, tissue samples were minced into 0.5–1 mm fragments and seeded into a ClinoReactor (CELVICO, Denmark). The GBM organoid culture medium was formulated as follows (32): 50% DMEM:F12 (Basal Media, China), 50% NeuroGro (Basal Media), 1× Glutamax (Thermo Fisher Scientific, USA), 1× NeaaS (Thermo Fisher Scientific), 1× N2 supplement (Basal Media), 1× B27 supplement (Basal Media), 1× 2‐mercaptoethanol (Thermo Fisher Scientific), and 2.5 mg mL^−1^ human insulin (Sigma, USA). Rotation was maintained at 25 rpm in a ClinoStar (CELVICO) culture chamber at 37 °C, 5% CO_2_, and 90% humidity. The culture medium was replaced every 48 h.

### GBO Growth Analysis and Total Cell Count

Similarly sized GBOs (diameter of 0.5–1 mm) were placed in low‐adhesion 96‐well plates to measure the GBO growth over time. Weekly observations and images were captured using an inverted microscope CKX31 (Nikon, Japan), and the diameter of each GBO was quantified using ImageJ software v 1.8.0. Growth rates were calculated relative to the original size for each time point. The measurements were performed on three individual GBOs per sample.

After measuring the diameter, the GBOs were dissociated into single cells, and cell numbers were determined using an automated cell counter, allowing for the calculation of the relationship between the GBO cell count and diameter.

### GBO Identification: Hematoxylin and Eosin Staining, mIHC, and Whole‐Exome Sequencing

GBOs and parental tumor tissue paraffin sections were prepared according to standard procedures, which included mIHC staining with the selected markers: GFAP for glioma cells, Ki67 for proliferation, Nestin, Olig2, BLBP, and SOX2 (ZEN‐BioScience, China) for stemness, B7‐H3, IL‐13Rα2 for target markers, CD206 for macrophages, and CD3 for T cells. Sections were counterstained with a DAPI‐containing anti‐fading mounting medium (Yeasen Biotechnology Co., Ltd., China). The Mantra quantitative pathology imaging analysis system captured images, and Inform Tissue Finder software was used for image processing and analysis. Perform Hematoxylin and Eosin Staining according to standard procedures.

The CTAB method was used to extract DNA from the GBOs and parental tumor tissues for whole‐exome sequencing. High‐quality samples (OD260/280 = 1.8–2.0, OD260/230 ≥ 2.0) were selected for library construction, with 0.5 µg of DNA used to build sequencing libraries. The TruSeq Nano DNA HT Sample Prep Kit (Illumina, USA) was used according to the manufacturer's instructions, and index codes were added to each sample.

Raw reads, including those with adapter contamination and unrecognizable nucleotides (N bases > 10), were trimmed using Fastp software.^[^ ^76^ ^]^ The trimmed reads were mapped to the reference using BWA‐MEM software.^[^ ^77^ ^]^

Aligned BAM files were sorted using the same tools according to the modified GATK Best Practice.^[^ ^78^ ^]^ Germline variant calling encompassing single nucleotide polymorphisms (SNPs) and insertions and deletions (INDELs) across all samples was performed using the HaplotypeCaller and GenotypeCaller programs in Sentieon Genomics Tools. Somatic variations were identified using the Mutect2 module in Sentieon Genomics Tools.

Structural variations were identified using Manta^[^ ^79^ ^]^ by considering the split alignments, soft clipping, insert size, paired alignments, and transchromosomal events.

CNVs were detected using a CNV kit,^[^ ^80^ ^]^ and the core window method was used for CNV detection.

Variants, including SNPs, structural variants, and INDELs, were categorized based on their positions on the chromosome, including intergenic exons, introns, regions, 1‐kb upstream and downstream regions, and untranslated regions. In addition, they were categorized based on their effects, such as start codon gain or loss, missense mutations, stop codon gain or loss, and splicing mutations.

### Dissociation of GBOs and scRNA‐seq

Upon harvesting, the GBOs were dissociated using a Miltenyi Tissue Dissociation Kit 2 (Germany). Cell count and viability were assessed using a fluorescence cell analyzer (Countstar, China) with the AO/PI reagent. Fresh cells were resuspended at a concentration of 1 × 10^6^ cells mL^−1^ in 1× phosphate‐buffered saline containing 0.04% bovine serum albumin (BSA).

ScRNA‐seq libraries were generated using the SeekOne® MM Single Cell 3′ library preparation kit (SeekGene, China). An appropriate number of cells were loaded into the flow channel of the SeekOne MM chip, settling into microwells through gravity. Cell‐barcoded magnetic beads (CBBs) were introduced into the flow channel and allowed to settle in microwells under the influence of a magnetic field. Subsequent cell lysis within the MM chip released the RNA, which CBBs captured in the same microwell. Reverse transcription was then performed at 37 °C for 30 min to label cDNA with a cell barcode on the beads. After Exonuclease I treatment, barcoded cDNA on the CBBs was hybridized with a random primer containing the SeqPrimer sequence at the 5′ end. The resulting second‐stranded DNA was denatured from the CBBs, purified, and amplified using PCR. The amplified cDNA product was subjected to full‐length sequencing adapter addition and sample indexing using indexed PCR. The indexed sequencing libraries were purified using SPRI beads, quantified using quantitative PCR (KAPA Biosystems KK4824), and sequenced on an Illumina NovaSeq 6000 with a PE150 read length or the DNBSEQ‐T7 platform with a PE100 read length.

Library size normalization for each cell was performed using Seurat's Normalize Data (v4.0.0).^[^ ^81^ ^]^ All libraries were integrated using FindIntegrationAnchors and IntegrateData with the default parameters. ScaleData was used to remove the variability in the number of UMIs. Subsequently, RunPCA, RunTSNE and RunUMAP were used for dimension reduction. FindAllMarkers compared each cluster with all the others to identify cluster‐specific marker genes. Retained marker genes had to be expressed in a minimum of 10% of cells, with a minimum log‐fold change threshold of 0.25. Differentially expressed genes in clustering were considered significant if the adjusted *P*‐value was <0.05 and avg_log2FC was ≥0. Clusters were annotated using cell type‐specific signatures and marker genes.

The CellPhoneDB Python package (v 2.1.7)^[^ ^82^ ^]^ was used to detect ligand–receptor interactions and predict communication among different cell types. Specific interactions classified by ligand or receptor expression were identified in >10% of cells within a cell type to focus on the most relevant interactions. Pairwise comparisons were conducted between cell types. The mean receptor and ligand expression levels in the interacting clusters were determined. A *P*‐value for the likelihood of cell‐type specificity of the corresponding ligand–receptor complex was obtained by calculating the proportion of means higher than the actual mean. Biologically relevant interactions were also identified.

### Construction of CAR and Lentivirus Production

Clinical‐grade lentiviral vectors were used. The CAR structure comprised CD19, B7‐H3, GD2‐specific single‐chain antibody or mutant IL‐13 (E13K.R66D; S69D.R109K)^[^ ^83^ ^]^ fragment linked by glycine/serine repeat connectors, a hinge domain, CD8 or CD28 transmembrane, 4‐1BB signaling domain as the co‐stimulatory molecule (or OX40 and 4‐1BB), and the CD3‐Zeta chain signaling domain of the T cell receptor (Figure 4B and Figure S7G, Supporting Information). The mouse PGK promoter was used to drive the expression of green fluorescent protein (GFP) sequence, which was a fluorescent label for the CAR. Sequences encoding the transgene were synthesized using the Genewiz Gene Synthesis Service and subcloned into the lentiviral backbone.^[^ ^84^ ^]^ Validation was performed using sequencing.

Viral production used a three‐plasmid system: the lentiviral plasmid encoding CAR, packaging plasmid pSPAX2 (AddGene, plasmid #12260), and pMD2.G (AddGene, plasmid #12259) were co‐transfected into HEK293T cells to generate lentiviral supernatants. Supernatants were collected at 36 and 60 h, filtered through a 0.45 µm filter to eliminate cell debris, and concentrated tenfold using centrifugation at 28000 rpm and 4 °C for 2 h. The resulting lentiviral particles were resuspended in pre‐cooled DMEM. Jurkat T cells were transduced to determine lentiviral titers. The collected viral particles were aliquoted and stored at −80 °C after concentration.

### γδ T Cell Isolation and Cultivation

Peripheral blood from healthy volunteers was density gradient centrifuged using Lymphoprep (STEMCELL Technologies, USA) at 20 °C to isolate peripheral blood mononuclear cells. Purified γδ T cells were separated using the Human TCR γ/δ T Cell Isolation Kit (Miltenyi, Germany), according to the manufacturer's instructions. Cells were resuspended in ImmunoSep Buffer (Miltenyi) and sequentially mixed with separation beads. γδ T cells were isolated using MS Separation columns (Miltenyi) with the MACS MultiStand magnetic stand. After purification, γδ T cells were resuspended at 1 × 10^6^ cells mL^−1^ in ImmunoCult‐XF T Cell Expansion Medium (Stem Cell Technologies). A similar protocol was used to isolate αβ T cells as a control.

Lymactin‐γδ T antibody (Baso, China) was added at 1 mL/20 mL of the culture medium to activate Vγ9Vδ2 T cells. The culture was maintained with the addition of 200 IU mL^−1^ of IL‐2 (Tianjin Hemay Source, China) or cytokine cocktail A (200 IU mL^−1^ IL‐2, 100 ng mL^−1^ IL‐4, 7 ng mL^−1^ IL‐21, 5 ng mL^−1^ transforming growth factor‐β (TGF‐β), and 70 ng mL^−1^ IFN‐γ, all from Tianjin Hemay Source, China). After two weeks, the Lymactin‐γδ T antibody was added again, and the culture was continued for an additional week.

ImmunoCult Human CD3/CD28/CD2 T Cell Activator (STEMCELL Technologies) was added at 25 µL/1 mL to the culture medium to activate Vδ1 T cells. The culture was maintained with the addition of 200 IU mL^−1^ IL‐2 or cytokine cocktail B (200 IU mL^−1^ IL‐2, 70 ng mL^−1^ IL‐15, 20 ng mL^−1^ IL‐7, 100 ng mL^−1^ IL‐4, and 7 ng mL^−1^ IL‐21). After two weeks, the T Cell Activator was added again, and the culture was continued for another week using 200 IU mL^−1^ of IL‐2 or cytokine cocktail C (70 ng mL^−1^ IL‐15, 50 ng mL^−1^ IL‐18).

CD3/CD28/CD2 T Cell Activator and 200 IU mL^−1^ of IL‐2 were added to the culture medium to activate and expand αβ T cells. All cell cultures were maintained at 37 °C and 5% CO_2_ with a cell density of 1 × 10^6^ cells mL^−1^.

### γδ T Cell Transduction

To prepare CAR‐γδ T cells, γδ T cells must first be activated to enhance their susceptibility to lentivirus infection. On the third day after activation, 1× NATE solution (InvivoGen, France) 10 µL was added to 1 mL of γδ T cells and incubated at 37 °C for 30 min. Subsequently, CAR lentiviruses (multiplicity of infection = 10) were added into the γδ T cells (2 × 10^5^ cells mL^−1^) to generate CAR‐γδ T cells. The cells were cultured with cytokines for 48 h. Transduced T cells were collected and cultured in a cytokine‐containing medium. The transduction efficiency was determined using flow cytometry analysis of CAR‐γδ T cell GFP expression. γδ T cells were counted using an automated cell counter (Countstar). αβ T cell transduction was performed following the same procedure.

### Flow Cytometry

Data were collected using a Novocyte flow cytometer (Agilent, Santa Clara, CA). Compensation was adjusted using OneComp eBeads (BD Biosciences, USA). Isotype controls (antibodies of the same species and class as the primary antibody) were used simultaneously. All samples were stained using a Live/Dead Cell Staining Kit (Life Technologies) prior to antibody staining. CAR‐γδ T cell transduction efficiency was determined based on GFP expression. The following antibodies were used for subset and functional analysis of γδ T cells: FITC anti‐human CD3, PE anti‐human TCR Vδ2, APC anti‐human CD223 (LAG‐3), PE anti‐human CD366 (TIM‐3), PE anti‐human CD314 (NKG2D), APC anti‐human Perforin, APC anti‐human CD69, PE anti‐human CD107a (LAMP‐1), all from BioLegend (USA), APC anti‐human Vδ1 (Miltenyi), and FITC anti‐human amphiregulin (AREG) (CUSABIO, China). For tumor cell lines and GBOs, APC anti‐human CD276 (B7‐H3) antibody (BioLegend) was used to assess B7‐H3 expression, and PE anti‐human CD213a2 (IL‐13Rα2) was used to analyze IL‐13Rα2 expression. APC anti‐human CD163 and PE anti‐human CD68 antibody (BioLegend) were used to detect the changes in macrophage phenotypes. Specific flow cytometry staining was performed according to the manufacturer's instructions. The cells were resuspended in DMEM for CD107a staining, and Cell Stimulation Cocktail (plus protein transport inhibitors) was added for stimulation and culture. After treatment with Fixation Buffer and Permeabilization Wash Buffer (Proteintech, USA), perforin‐stained cells were analyzed using flow cytometry. Data were analyzed using NovoExpress 1.5.6 (Agilent, USA) or FlowJo software (Tree Star, Ashland).

### Long‐Term Proliferation Assay

On day 15, CAR‐γδ T cells activated and expanded with a T cell activator, or Lymactin‐γδ T antibodies were co‐cultured with Raji cells at a 1:1 ratio for 7 d. Without the addition of cytokines, the cell density was maintained at 1 × 10^6^ cells mL^−1^, and fresh culture medium and target cells were replenished every 2–3 d. Cells were regularly collected, and T cell proliferation was analyzed using an automated cell counter.

### Co‐Culture–Cytokine Levels, CAR‐γδ T Cell Phenotype, and Cytotoxicity Assay

Raji‐luc cells were seeded at a ratio of 1:1 in a 96‐well plate (10^4^ cells/well), and CAR‐γδ T cells or controls were added. To detect cytokine secretion, the cell culture supernatant was collected after 48 h of co‐culturing, and a Human Cytokine Cytometric Bead Array (CBA) Kit (BD Biosciences) was used to measure cytokines (IL‐1β, IL‐2, IL‐4, IL‐5, IL‐6, IL‐8, IL‐10, IL‐12p70, IL‐17, IFN‐γ, TNF‐α, and IFN‐α). Data were analyzed using the FCAP Array v3.0 software (BD Biosciences) following the manufacturer's instructions. Flow cytometry analysis of the CAR‐γδ T cell phenotype before and after tumor cell killing was performed as described above. For cytotoxicity assays, d‐luciferin (40 µL, 150 µg mL^−1^) was added after 12 h of co‐culture, and bioluminescence imaging was conducted using the IVIS Spectrum (PerkinElmer, USA). Data were analyzed using Spectrum Living Images 4.0. Raji cells pre‐stained with 1,1′‐dioctadecyl‐3,3,3′,3′‐tetramethylindodicarbocyanine perchlorate (DID) (Yeasen Biotechnology Co., Ltd.) were co‐cultured with γδ T cells for 4 h, and PI (Yeasen Biotechnology Co., Ltd.) was added for subsequent flow cytometry detection.

### Spheroid 3D Cell Culture

LN229 cells grown to a healthy state in traditional 2D cell culture plates were seeded into a pre‐wetted ClinoReactor (CELVIVO, Denmark) after reaching confluence. The culture was rotated at 25 rpm in a ClinoStar culture chamber (CELVIVO). Spheroids were cultured in humidified air at 37 °C with 5% CO_2_ for a minimum of 15 d, and the medium was changed every 2–3 d. Selected spheroids were transferred to culture dishes, and Z‐stack scans were performed using an All‐in‐One fluorescence microscopy system (BZ‐X, Keyence, Japan) to assess spheroid quality (density and roundness). The cell viability was analyzed using the Hybrid Cell Count Module (Keyence) after AO/PI (Countstar) staining.

2D‐cultured LN229 cells were stained with PE anti‐human IL‐13Rα2 and APC anti‐human B7‐H3, followed by flow cytometry to detect target protein expression. For 3D LN229 spheroids, Z‐stack scans were performed using the All‐in‐One fluorescence microscopy system after immunofluorescent double staining for B7‐H3 and IL‐13Rα2, and target protein expression was quantified using the Hybrid Cell Count Module.

### Double CAR‐γδ T Cell Cytotoxicity against LN229 Spheroids

LN229 spheroids with a diameter >100 µm were selected using a microscope pre‐stained with Hoechst (Yeasen Biotechnology Co., Ltd.), and six spheroids per well were seeded into a 96‐well plate. DIO (Yeasen Biotechnology Co., Ltd.)‐pre‐stained CAR‐γδ T cells or controls were added at an effector/target ratio of 1:1. PI staining was performed at 24 and 48 h, followed by Z‐stack scans using an All‐in‐One fluorescence microscopy system. Cell viability (the proportion of PI‐positive cells) was analyzed using a Hybrid Cell Count Module. Spheroid cell counts were determined by selecting spheroids with known diameters (>100 µm), digesting them into single cells with trypsin, counting the cells, and establishing a diameter–cell count relationship.

### γδ T and αβ T Cell Infiltration into GBO Samples

γδ and αβ T cells were co‐cultured with GBOs at an effector‐to‐target ratio of 1:1 (γδ and αβ T cells were cultured from the same donor batch) to assess the infiltration of γδ and αβ T cells into GBOs. T cells pre‐labeled with PKH‐26 or DIO (MedChemExpress, USA) were co‐incubated with GBOs in four‐well chambered culture plates (Thermo Fisher Scientific), and GBOs were pre‐labeled with Hoechst. Each well contained three similarly sized GBOs. Continuous imaging for 24 h was performed using a CQ1 high‐content imaging analysis system (Yokogawa, Japan), and the data were analyzed. Multi‐color fluorescent images were collected and analyzed at 24 and 48 h.

Different CAR‐transduced γδ T cells were co‐cultured with GBOs at a 1:1 ratio to investigate multiple CAR‐γδ T cell infiltration into GBOs. DIO‐pre‐labeled γδ T cells were co‐cultured with GBOs in U‐bottom 96‐well plates (Corning, USA). Images were acquired as previously described. CAR‐γδ T cell infiltration was analyzed using a region‐based approach. Concentric circles were drawn around the central point of GBOs at 50 µm intervals. Points were randomly selected on the concentric circle trajectory to draw circles with a diameter of 50 µm. The proportion of CAR‐γδ T cells within each circle was calculated using the Hybrid Cell Count Module.

### Heterogeneous B7‐H3/IL‐13Rα2 Expression in GBO Samples

Different GBO samples were selected, and B7‐H3 and IL‐13Rα2 antibodies were used to stain GBOs. Z‐stack scanning was performed using an All‐in‐One fluorescence microscopy system, and multi‐color fluorescence analysis of the acquired images was conducted using the Hybrid Cell Count Module.

### γδ T Cell Phenotypic and Cytokine Analyses and B7‐H3/IL‐13Rα2 Expression

Cell and culture medium samples were collected 48 h after culture under the experimental conditions mentioned above. For cell samples, the phenotype of CAR‐γδ T cells after tumor cell killing was analyzed using the flow cytometry protocol described earlier. Culture medium samples were rapidly frozen in liquid nitrogen and stored at −80 °C. The Human CBA Kit was used to detect cytokines, and analysis was conducted using FCAP Array software v3.0, following the manufacturer's instructions.

After co‐culture, GBO samples were obtained, and overall staining of GBOs was performed using antibodies against B7‐H3 and IL‐13Rα2. Images were obtained as previously described, and multi‐color fluorescence analysis was performed on the acquired images.

### In Vitro Co‐Culture of Multiple CAR‐γδ T Cells with GBOs

CAR‐γδ T cells and GBOs were co‐cultured at an effector‐to‐target ratio of 1:1 to further investigate the cytotoxic effects of double CAR‐γδ T cells on GBOs, B7‐H3 and IL‐13Rα2 CAR‐γδ T cells. Co‐cultivation was performed in ultralow attachment 24‐well plates (Corning, USA) with approximately six similarly‐sized GBOs per well (pre‐labeled with Hoechst). Co‐cultures were maintained in a CO_2_ incubator at 37 °C, 5% CO_2_, and 90% humidity. Co‐culture experiments were conducted using three GBO samples. PI was added to the GBOs and CAR‐γδ T co‐culture system at 24 and 48 h. Z‐stack scanning was performed using an All‐in‐One fluorescence microscope (Keyence). The acquired images were subjected to multi‐color fluorescence analysis using a Hybrid Cell Count Module (Keyence). Simultaneously, an unstained control group was established within the same series, and specimens were collected 48 h after culturing for multi‐color fluorescence immunohistochemical analysis.

The cytotoxic effects of triple CAR‐γδ T cells on GBOs were assessed based on the co‐cultivation of double CAR‐γδ T cells and GBOs mentioned above. B7‐H3, IL‐13Rα2, and GD2 target antigen expression in GBOs was determined. Then, CAR‐γδ T cells specific to B7‐H3, IL‐13Rα2, and GD2 were prepared as previously described, and their respective transduction efficiencies were assessed using flow cytometry. Finally, the triple CAR‐γδ T cells were co‐cultivated with GBOs, and the cytotoxicity was measured using the same methods.

### Rational Multiple CAR‐Vδ1 T Cell Therapy via Orthotopic Xenografting of GBO‐luc Animal Model In Vivo

Female NCG mice (NOD/ShiLtJGpt‐Prkdcem26Cd52Il2rgem26Cd22/Gpt, Nanjing GemPharmatech Co., Ltd.) at 6–8 weeks old were maintained in a specific pathogen‐free (SPF) environment.

To establish an orthotopic xenograft model of GBM, NCG mice were anesthetized using a gas anesthesia system. The right caudate nucleus of the mouse brain was chosen as the inoculation site with coordinates at 0.5 mm anterior to the bregma and 2.2 mm to the right in the sagittal plane. GBOs expressing GFP‐luciferase (luc) were generated via lentiviral infection. The GBO‐luc with confirmed heterogeneous expression of B7‐H3 and IL‐13Rα2 (with a total cell count of ≈5 × 10⁵) was inoculated into the caudate nucleus area.

To monitor tumor growth, mice were anesthetized with isoflurane, and 100 µL (3 mg) of D‐luciferin sodium salt (Yeasen Biotechnology Co., Ltd., China) was intraperitoneally injected. Ten minutes post‐injection, in vivo bioluminescence imaging was conducted using IVIS Spectrum. Images were acquired and quantified using Living Image software.

One week later, after confirming the successful establishment of the model via IVIS Spectrum, mice were randomly assigned to control, treatment, and combination treatment groups. Subsequently, 5 × 10⁵ multiple CAR‐Vδ1 T cells were stereotactically injected into the tumor site. Tumor volume was monitored weekly using IVIS Spectrum. Evaluation was performed using the GvHD scoring system, considering weight loss, activity, posture, fur texture, and skin integrity, each with scores of 0 for normal, 1 for mild, and 2 for moderate to severe, resulting in a maximum score of 10 and a minimum score of 0.

At the experimental endpoint, mice were euthanized, and their liver, kidney, spleen, heart, and lungs were collected and fixed in 10% formalin. After standard procedures, tissues were embedded in paraffin, and 5‐µm sections were prepared for H&E staining. Digital pathology scanners (Nanozoomer Digital Pathology scanner, Japan) were used to scan the slides at 20× magnification. H&E‐stained slides were blindly analyzed by two pathologists, and five images were randomly selected from each sample for pathological evaluation. After collecting the mouse brains, the tumor tissues were isolated and digested into single cells using Accutase (Yeasen Biotechnology Co., Ltd., China). Then, according to the standard flow cytometry procedure, the cells were stained with anti‐human TCR γδ, CD69, NKG2D, TIM‐3, and PD‐1 antibody (BioLegend). The data were analyzed using NovoExpress 1.5.6 (Agilent, USA) software.

### Statistical Analysis

Data from cell proliferation, cytotoxicity assays, cytokine detection, and survival analyses were summarized using descriptive statistical methods. Statistical analyses were performed using GraphPad Prism software v 7.0. Data are presented as the mean ± standard deviation (SD). Normality and homogeneity of variance were assessed for all quantitative data. All data represent at least three independent experiments unless otherwise specified. Quantitative analysis of immunohistochemistry was conducted in parallel, and fluorescence or confocal microscopy imaging was performed using identical settings. Statistical tests such as the Student's *t*‐test, analysis of variance, were used as appropriate. The log‐rank test was used for survival analysis. Statistical significance was set at *P* < 0.05. The notations *P < 0.05, **P < 0.01, ***P < 0.001, ****P < 0.0001, and nonsignificant (NS, *P* > 0.05) were used to indicate the significance level.

## Conflict of Interest

The authors declare no conflict of interest.

## Author Contributions

G.Z.: data curation, formal analysis, investigation, methodology, original draft, review & editing. Z.S.: formal analysis, funding acquisition, review & editing. Y.L.: investigation, visualization. J.L.: investigation, visualization. L.G.: formal analysis, resources. G.P.: methodology, resources. Y.J.: data curation, resources. B.M.: data curation, resources. Z.L.: methodology, resources. P.Z.: data curation, resources. D.T.: conceptualization, funding acquisition, project administration, supervision, review & editing. W.Z.: conceptualization, formal analysis, methodology, project administration, review & editing. C.W.: project administration, funding acquisition, resources, supervision, review & editing.

## Supporting information



Supporting Information

Supplemental Movie 1

## Data Availability

Research data are not shared.
