# Supplementary material for: Rational Design and Organoid‐Based Evaluation of a Cocktail CAR‐γδ T Cell Therapy for Heterogeneous Glioblastoma
Source: Adv Sci (Weinh). 2025 Mar 20;12(19):2501772. doi: 10.1002/advs.202501772 (PMC12097020; doi:10.1002/advs.202501772)
Supplement: Supplementary file 1 — Supporting Information [file ADVS-12-2501772-s001.docx]

**Title: Rational design and organoid-based evaluation of a cocktail CAR-γδ T cell therapy for heterogeneous glioblastoma**

**SUPPLEMENTARY MATERIAL**

**Supplementary Figures:** **
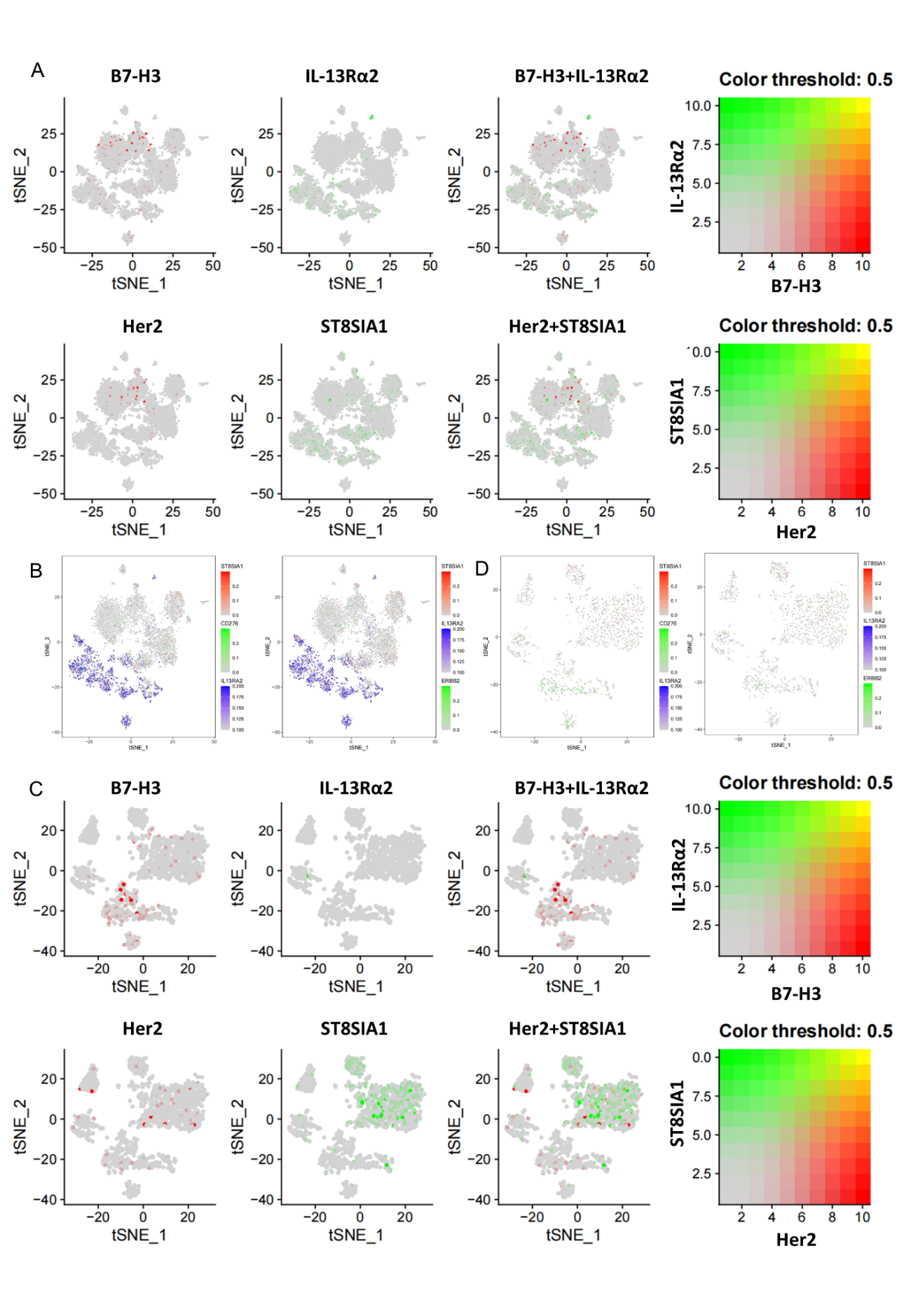
**

**Fig. S1. Analysis of public scRNA-seq datasets reveals heterogeneous gene expression in GBM and indicates that the screened gene combinations can improve coverage of tumor cell populations.**

(**A-B**) The tSNE plot demonstrates the expression of single target-gene, two target-gene, and three target-gene combinations within GBM cell populations (GSE138794).

(**C-D**) The tSNE plot demonstrates the expression of single target-gene, two target-gene, and three target-gene combinations within GBM cell populations (GSE140819).

**
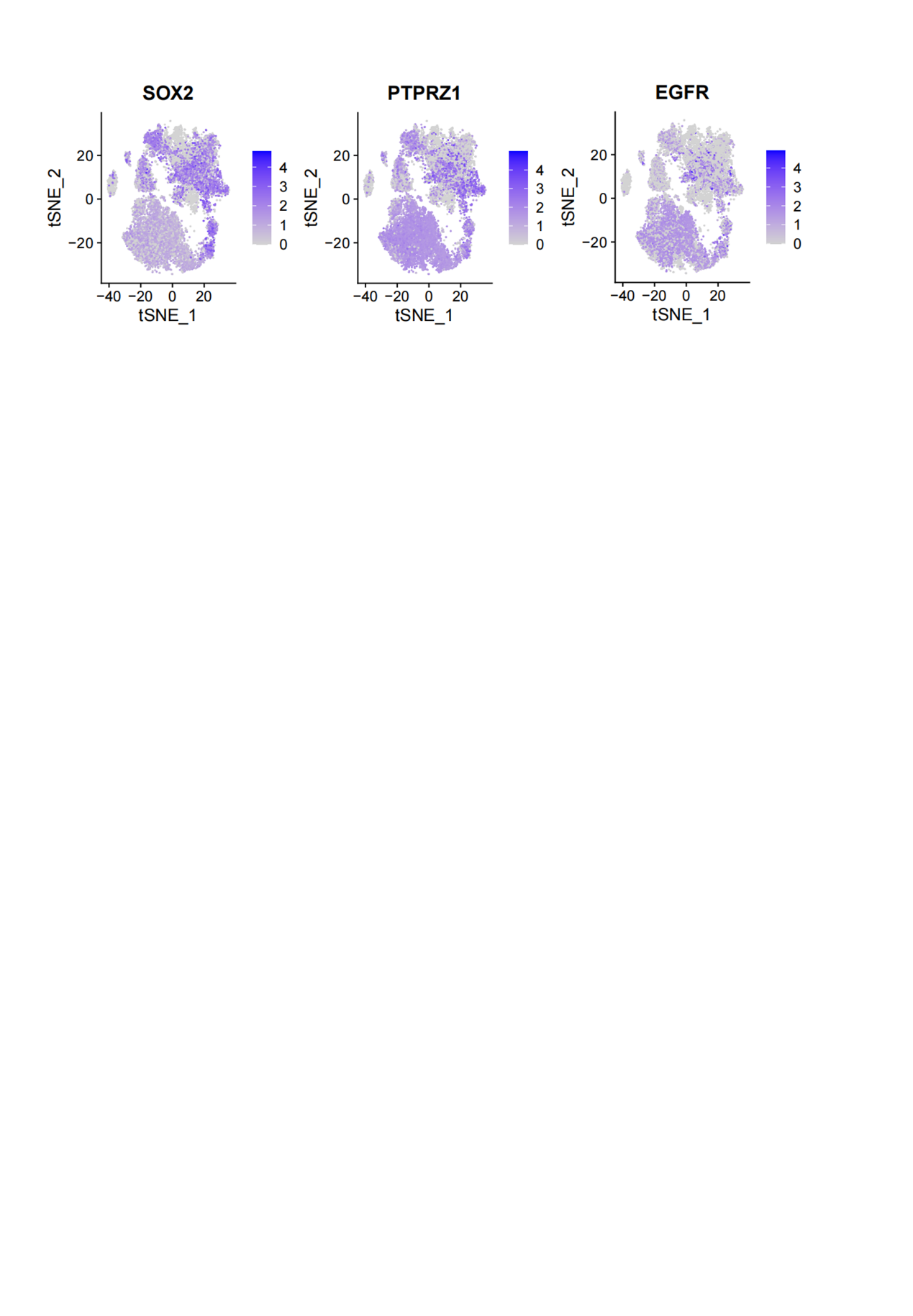
**

**Fig. S2.** The tSNE plot shows the expression of malignant genes in the selected samples.

**
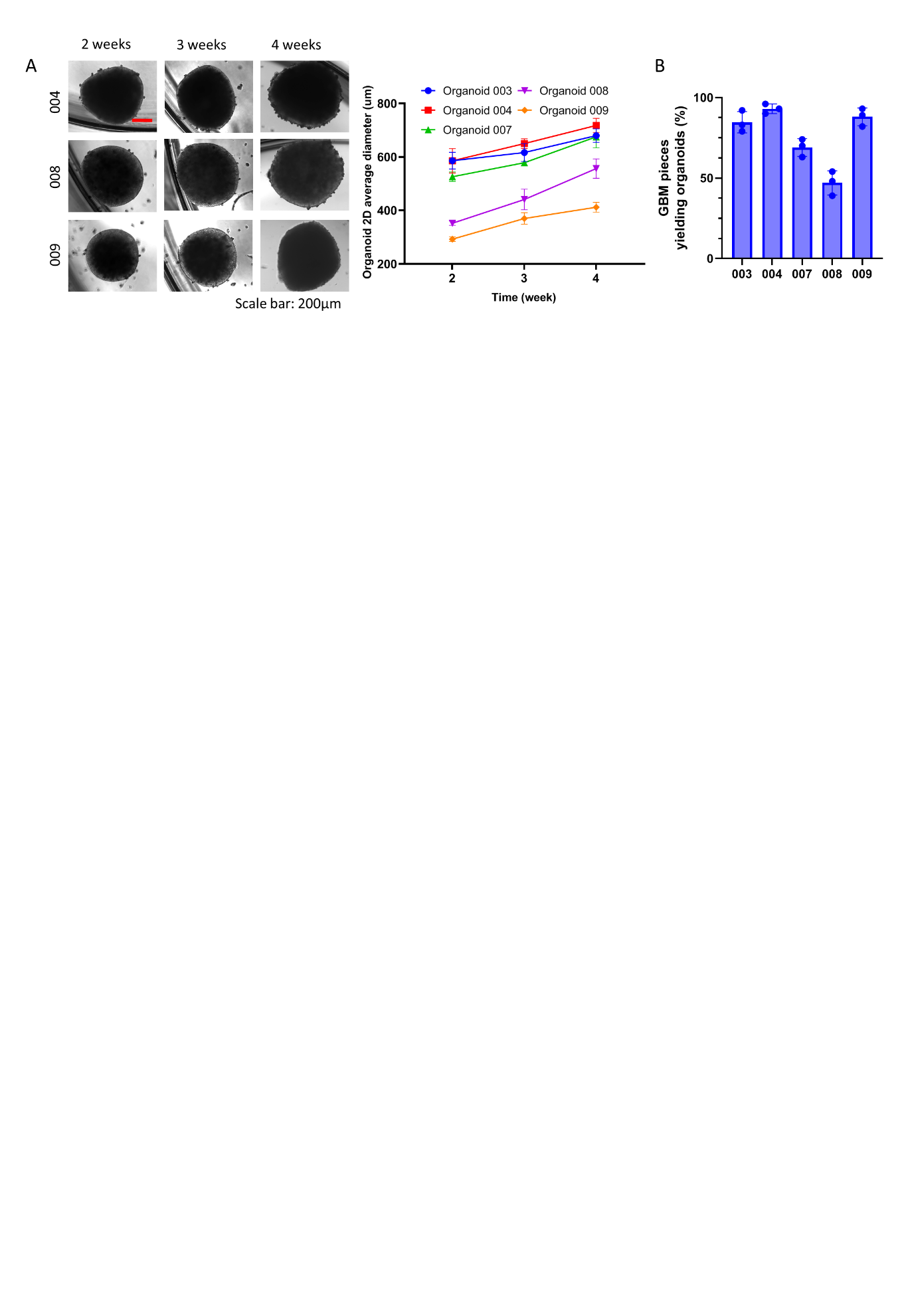
**

**Fig. S3.**  **Quantitatively measured to track GBOs development**

(**A**) Time series showing changes in the size of the same GBOs over time. 2D diameter of each GBO was measured and quantified (n = 6, three GBOs per sample). Data are presented as the mean ± SD of triplicate wells.

(**B**) Quantification of fragments from different tumors and generated GBOs after 2 weeks of cultivation. Data are presented as the mean ± SD (n = 5).


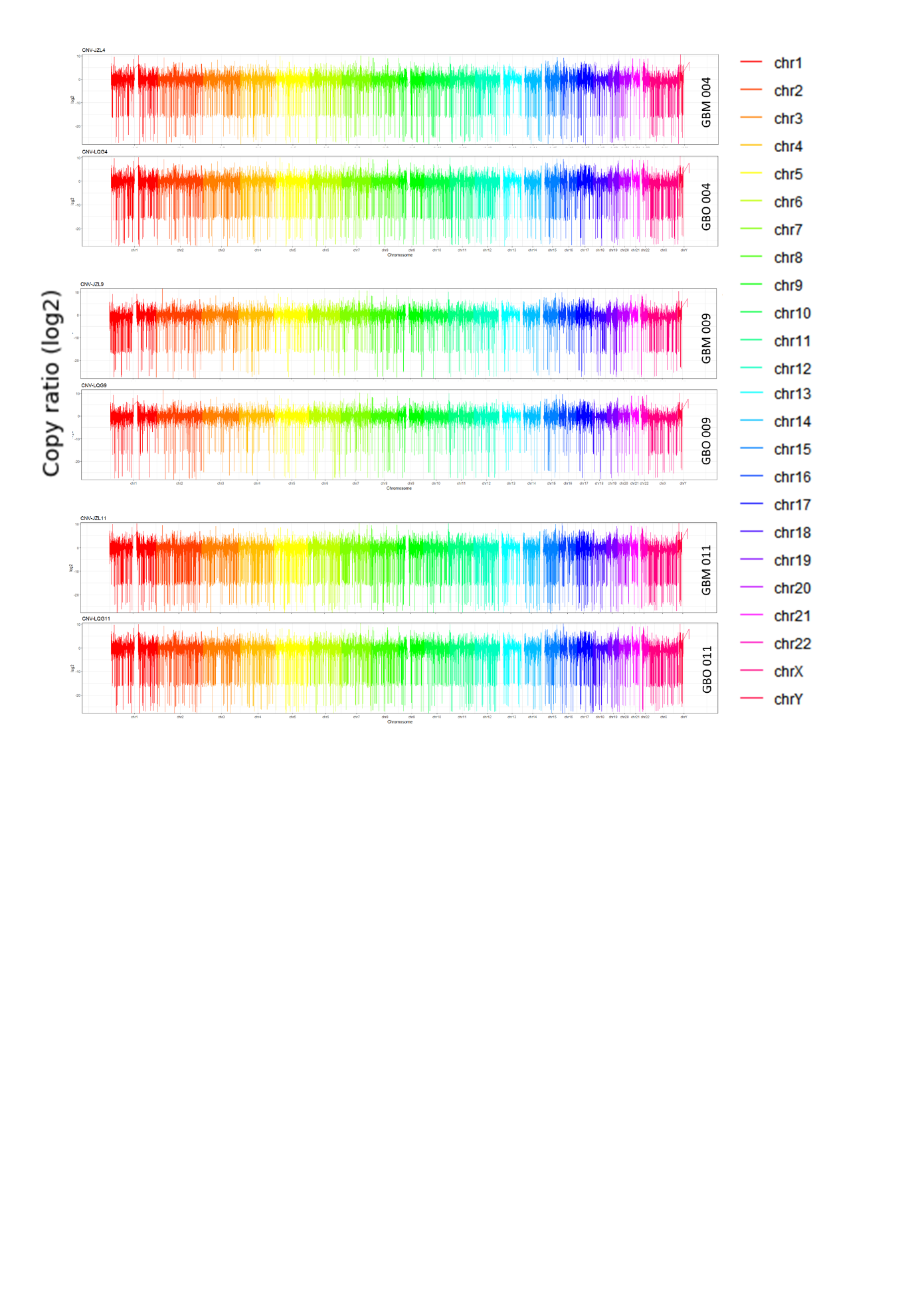


**Fig. S4. Manhattan plot showing the consistency of chromosomal CNVs between GBOs and parental GBM**

**
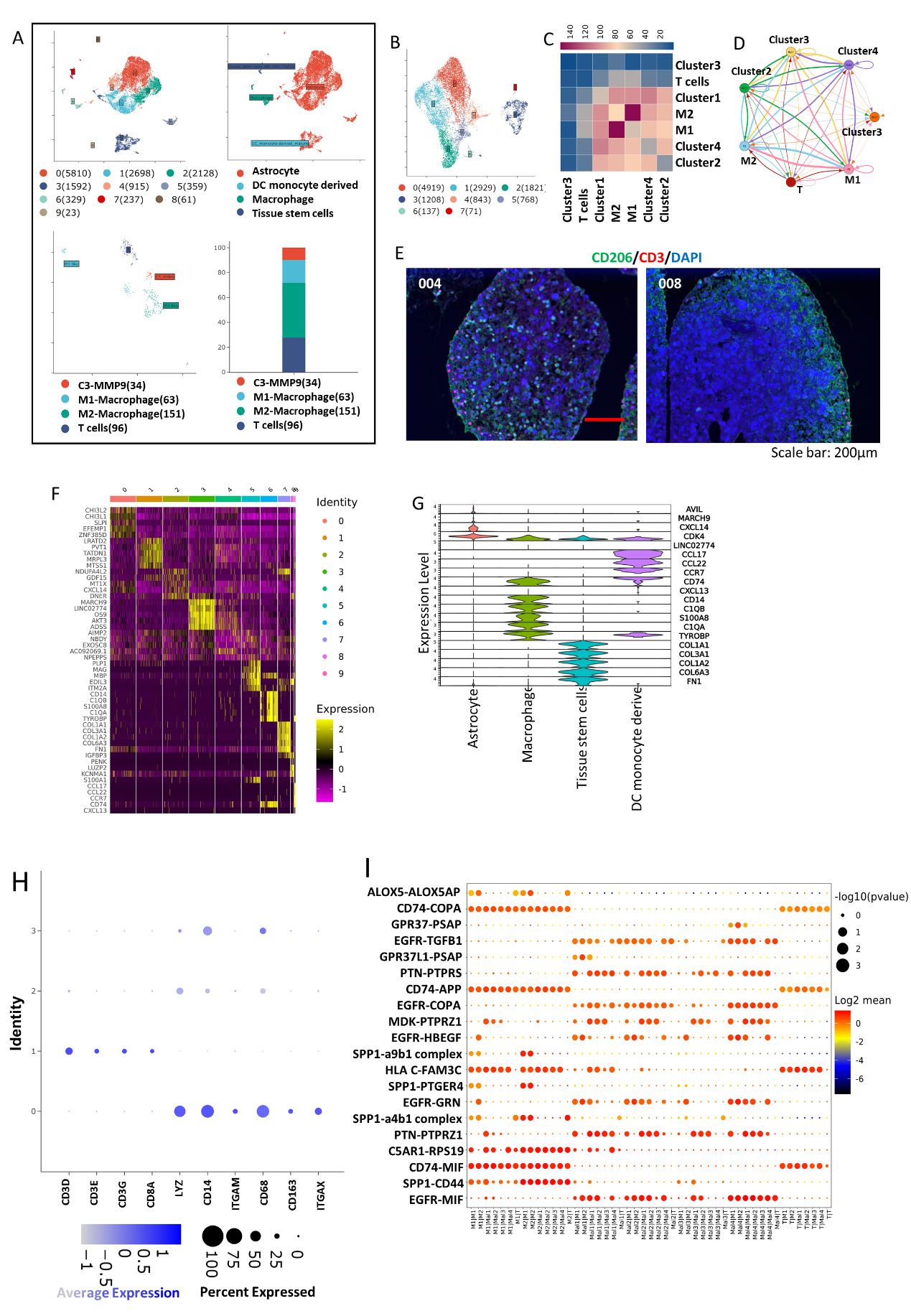
**

**Fig. S5. ScRNA-seq showing the cell cluster s in the microenvironment of GBO and cell cluster-specific marker gene expression**

(**A**) UMAP plot showing clustering and coloring of GBO samples, scRNA-seq expression in GBO samples, clustering and coloring of macrophage-like subgroups in GBO samples, and the proportion of macrophage and T cell subgroups in the macrophage-like subgroups.

(**B**) UMAP plot showing the re-clustering and coloring of tumor cell subgroups in GBO samples based on proliferation-related gene expression.

(**C, D**) Heatmap and network plot displaying communication relationships between different cell subgroups after artificially merging seven cell subgroups into four cell subgroups.

(**E**) Multi-color immunohistochemistry displaying CD206 and CD3 expression in GBOs.

(**F**) Heatmap displaying cell cluster-specific marker gene expression in GBO samples.

(**G**) Violin plot showing cell cluster-specific marker gene expression in GBO samples for tumor and non-tumor cells.

(**H**) Bubble plot of non-tumor cell cluster-specific marker gene expression in GBO samples.

(**I**) Bubble plot showing interactions between various cell subgroups in GBO samples. The top 20 receptor–ligand pairs based on average and differential expression were selected for display.

**
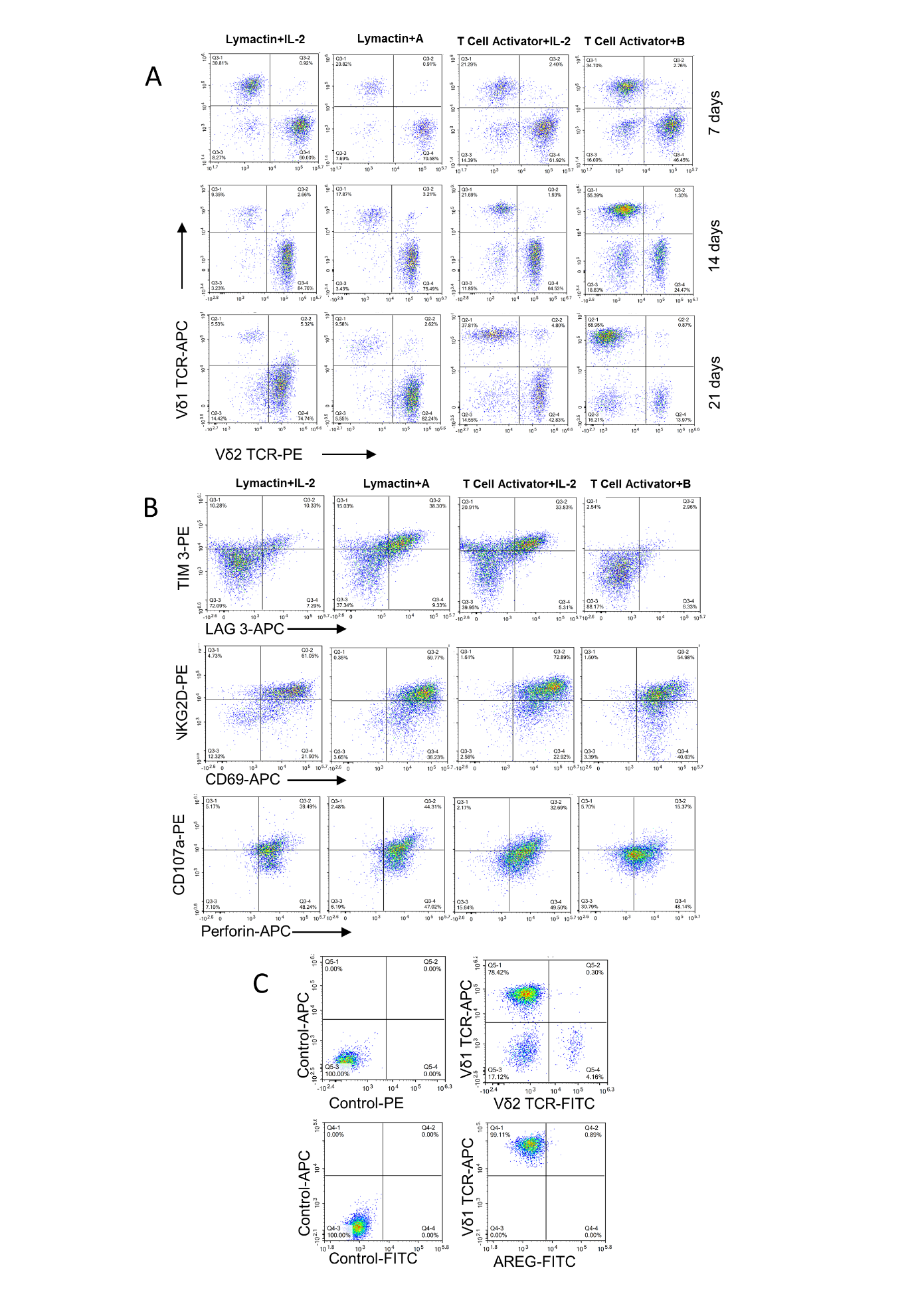
**

**Fig. S6. Flow cytometry detection of functional phenotypes in γδ T cells**

(**A**) Changes in Vδ1 and Vδ2 T cell subtypes were monitored weekly during the 21-day cultivation period. Lymactin-activated γδ T cells were predominantly of the Vδ2 subtype, referred to as Vδ2 T cells. γδ T cells activated by a T cell activator were mainly of the Vδ1 subtype, named Vδ1 T cells (Expansion of Fig. 4C).

(**B**) Flow cytometry analysis of harvested γδ T cells after 21 days of cultivation. Representative flow cytometry images of the exhaustion (TIM3, LAG3) and cytotoxic phenotypes (NKG2D, CD69, CD107a, and perforin) of γδ T cells.

(**C**) Flow cytometry analysis of Vδ1 T cells showing almost no AREG phenotype.

**
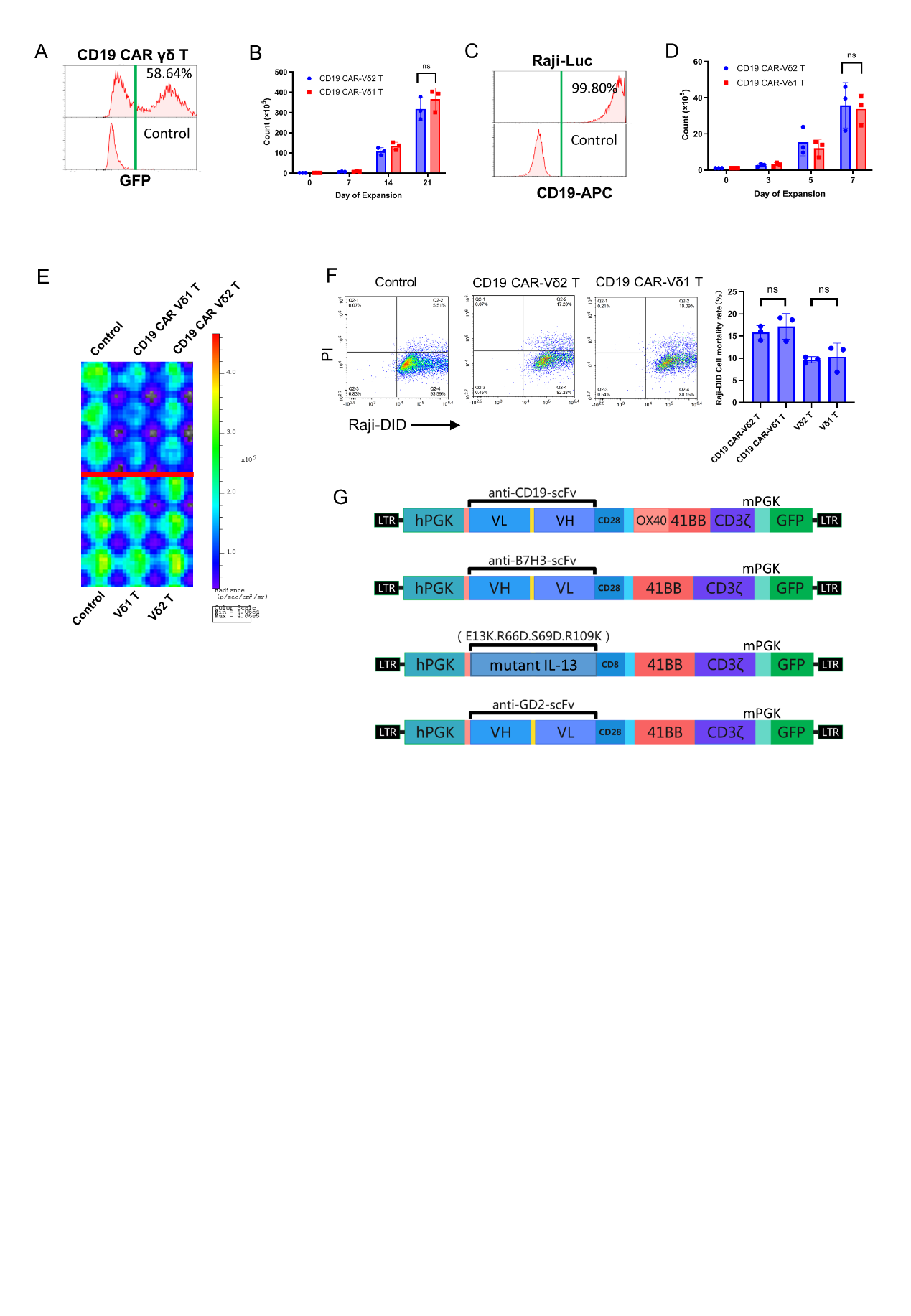
**

**Fig. S7. Transduction efficiency of CD19 CAR-γδ T cells and their cytotoxic effect on Raji cells**

(**A**) γδ T cells transduced with CD19 CAR lentivirus have a high transduction efficiency.

(**B**) CD19 CAR-γδ T cells exhibit vigorous proliferation activity. After the 21-day expansion process, nearly 400-fold proliferation was achieved (n = 3). NS, P > 0.05 using the Student’s t-test.

(**C**) Flow cytometry detection shows widespread CD19 antigen expression in Raji cells.

(**D**) CD19 CAR-γδ T cells co-cultured with CD19-expressing Raji cells for 7 days exhibit long-term proliferation activity (n = 3). NS, P > 0.05 using the Student’s t-test.

(**E**) Bioluminescence detection 12 h after co-culturing CD19 CAR-γδ T with Raji-luc cells shows a significant cytotoxic effect (n = 3).

(**F**) Flow cytometry detection of the proportion of dead cells (PI) 4 h after co-culturing CD19 CAR-γδ T with Raji cells pre-stained with DID cell membrane dye (n = 3). NS, P > 0.05 using one-way analysis of variance with Tukey’s post-hoc test.

(**G**) Schematic of the CD19, B7-H3, IL-13Rα2, and GD2 CAR structures


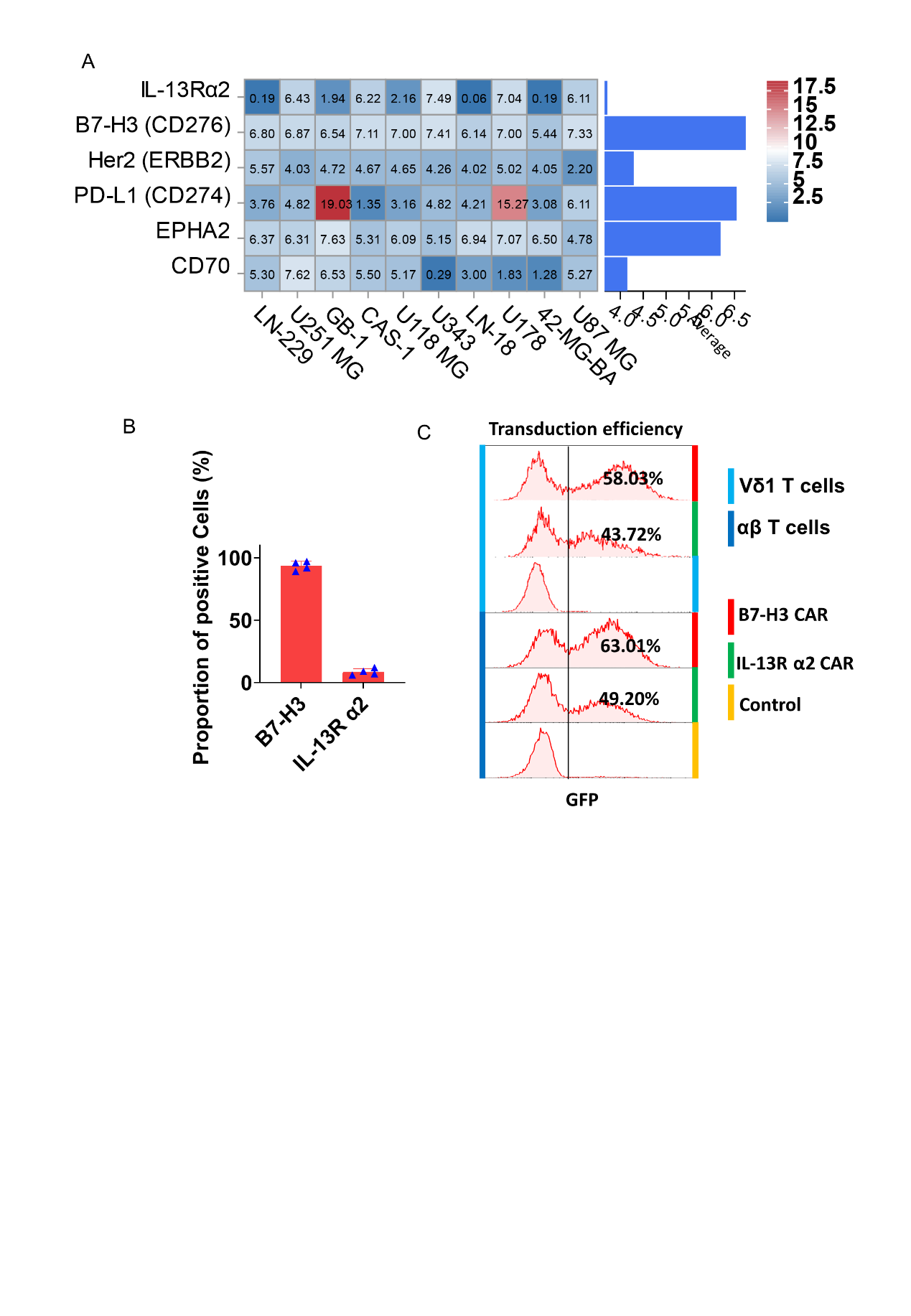


**Fig. S8. Expression levels of different target genes in commonly used glioma cell lines and target expression of LN229 cells, and transduction efficiency of multiple CAR-γδ T cells and therapeutic effects**

(**A**) Expression distribution of different genes in different cell lines. The y-axis represents gene expression, and the x-axis represents different cell lines. Different colors represent expression levels.

(**B**) Quantitative analysis of B7-H3 and IL-13Rα2 expression in LN229 cells using flow cytometry.

(**C**) Representative images showing transduction efficiency using flow cytometry.


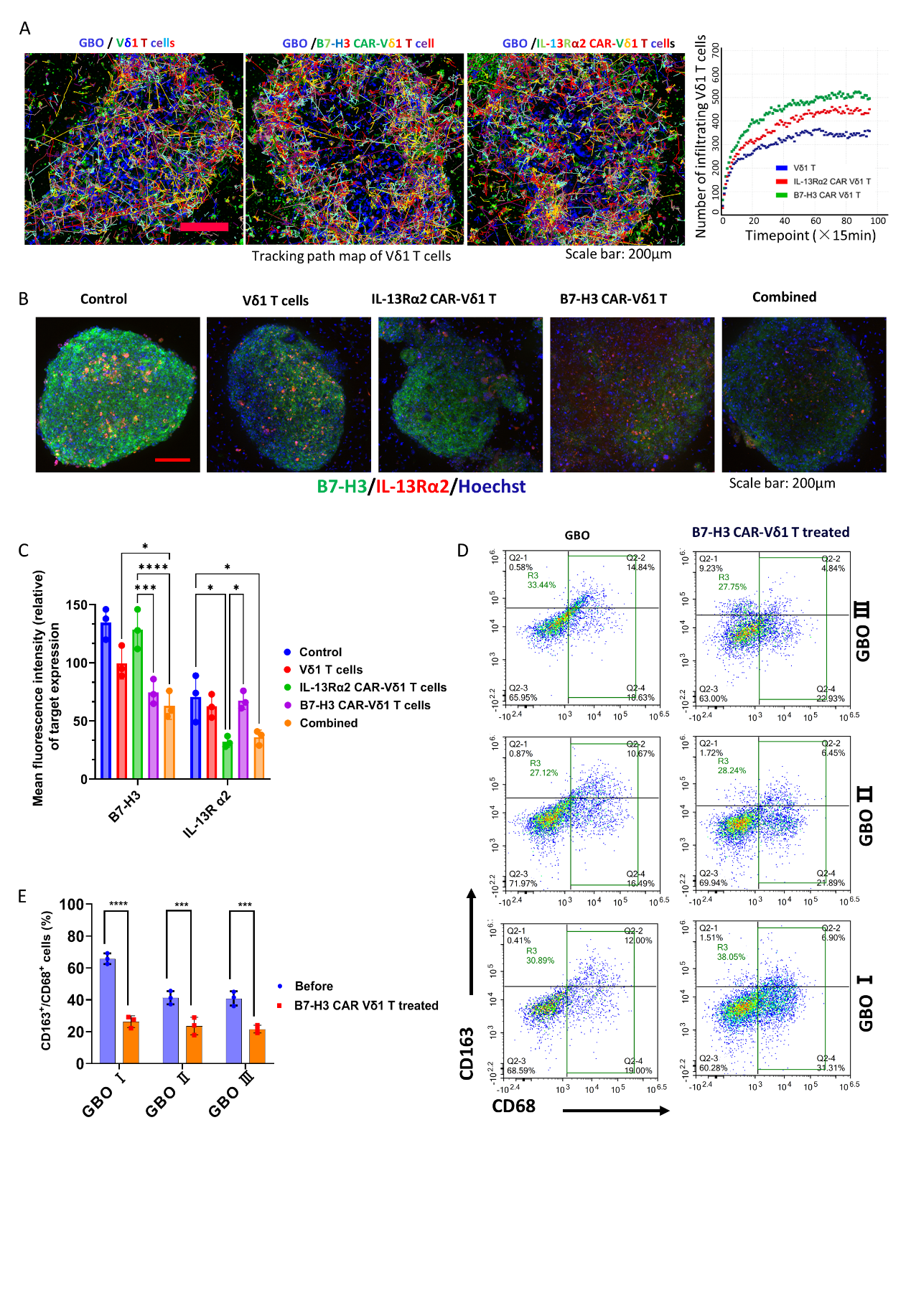


**Fig. S9. Similar tissue infiltration exhibited by Vδ1 T cells engineered to target different antigens and effective clearance of antigen-positive cells by double CAR-γδ T cells.**

(**A**) Transduced and non-transduced Vδ1 T cells exhibit high infiltration levels and quantifiable infiltrating cell numbers in GBOs. Blue represents GBOs (Hoechst), and the gradient color represents Vδ1 T cells. Scale bar: 200 μm.

(**B, C**) Fluorescence intensity of B7-H3 or IL-13Rα2-positive cells in GBOs significantly decreases after combination therapy with double CAR-γδ T cells. Blue represents GBOs (Hoechst), and green represents B7-H3-APC. Red represents IL-13Rα2-PE. Scale bar: 200 μm. Data are presented as the mean ± SD of triplicate wells. *P < 0.05, ***P < 0.001, ****P < 0.0001 using one-way analysis of variance with Tukey’s post-hoc test.

**(D, E)** Flow cytometry was used to detect the changes in macrophage phenotypes after the infiltration of B7-H3 CAR-Vδ1 T cells into GBOs (n = 3). ***P < 0.001, ****P < 0.0001 as determined by the Student’s t-test.


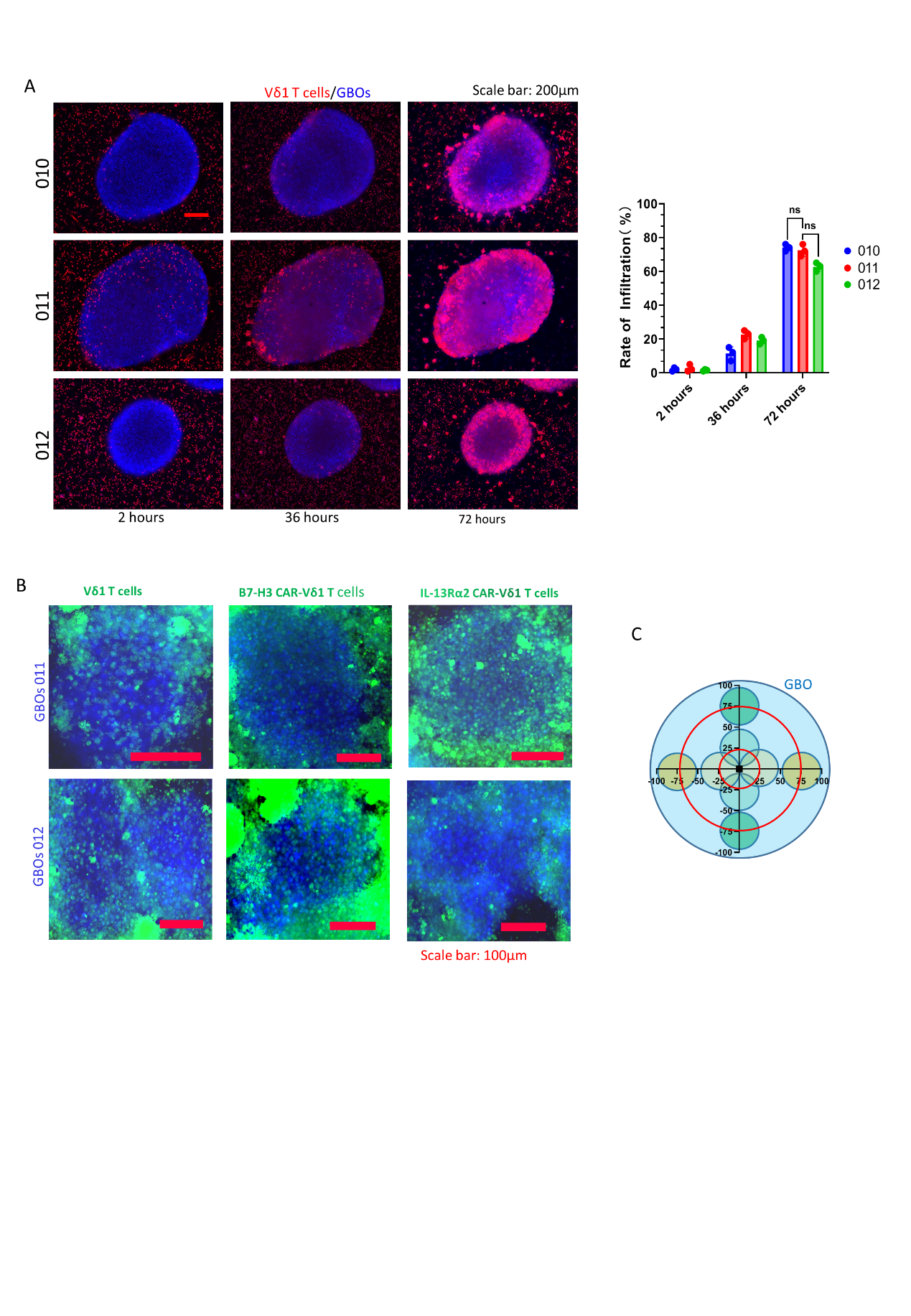


**Fig. S10. Dynamic and static analysis of Vδ1 T cell infiltration into GBOs**

(**A**) Vδ1 T cells exhibit consistent infiltration and proliferative capacity in different GBOs (n = 3). Blue: GBOs (Hoechst), red: Vδ1 T cells (PKH-26). Scale bar: 200 μm. NS, P > 0.05 using one-way analysis of variance with Tukey’s post-hoc test.

(**B**) Vδ1 T, B7-H3 CAR-Vδ1 T, and IL-13Rα2 CAR-Vδ1 T cells exhibit similar GBO infiltration. Blue: GBOs (Hoechst), green: Vδ1 T cells (DIO). Scale bar: 100 μm.

(**C**) Region-based analysis of Vδ1 T cell infiltration patterns.


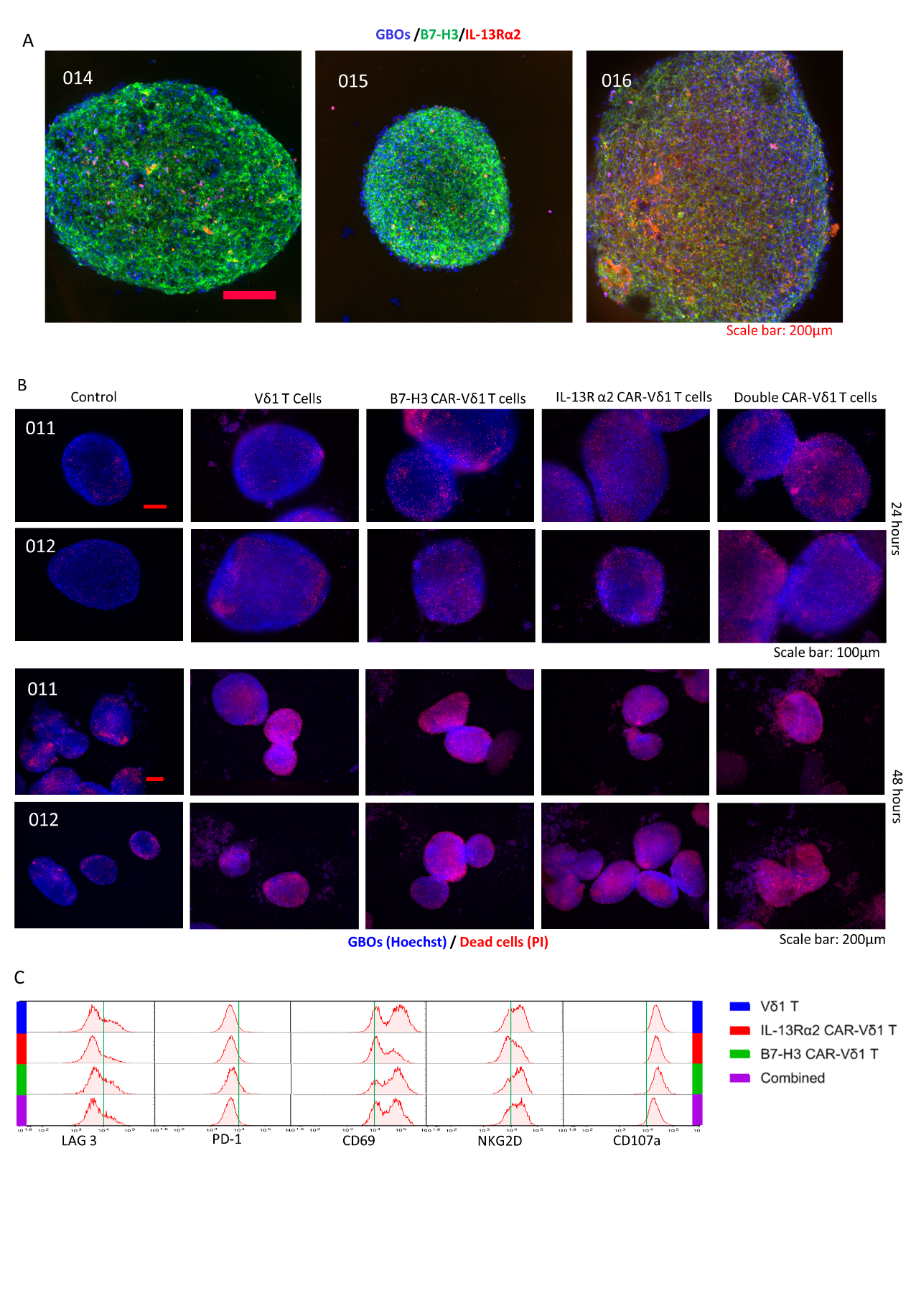


**Fig. S11. Heterogeneity among GBOs from different patients and the efficacy of multiple (double) combination therapy for GBOs from diverse sources**

(**A**) Immunofluorescence staining showing the heterogeneous B7-H3 and IL-13Rα2 expression in GBO samples, both intra- and intertumor. Scale bar: 200 μm.

(**B**) Efficacy of double CAR-γδ T cell immunotherapy on GBOs from different tumor sources.

(**C**) Flow cytometry detected γδ T cells after co-culturing with GBOs for 48 h, revealing a phenotype characterized by low exhaustion and high cytotoxicity.


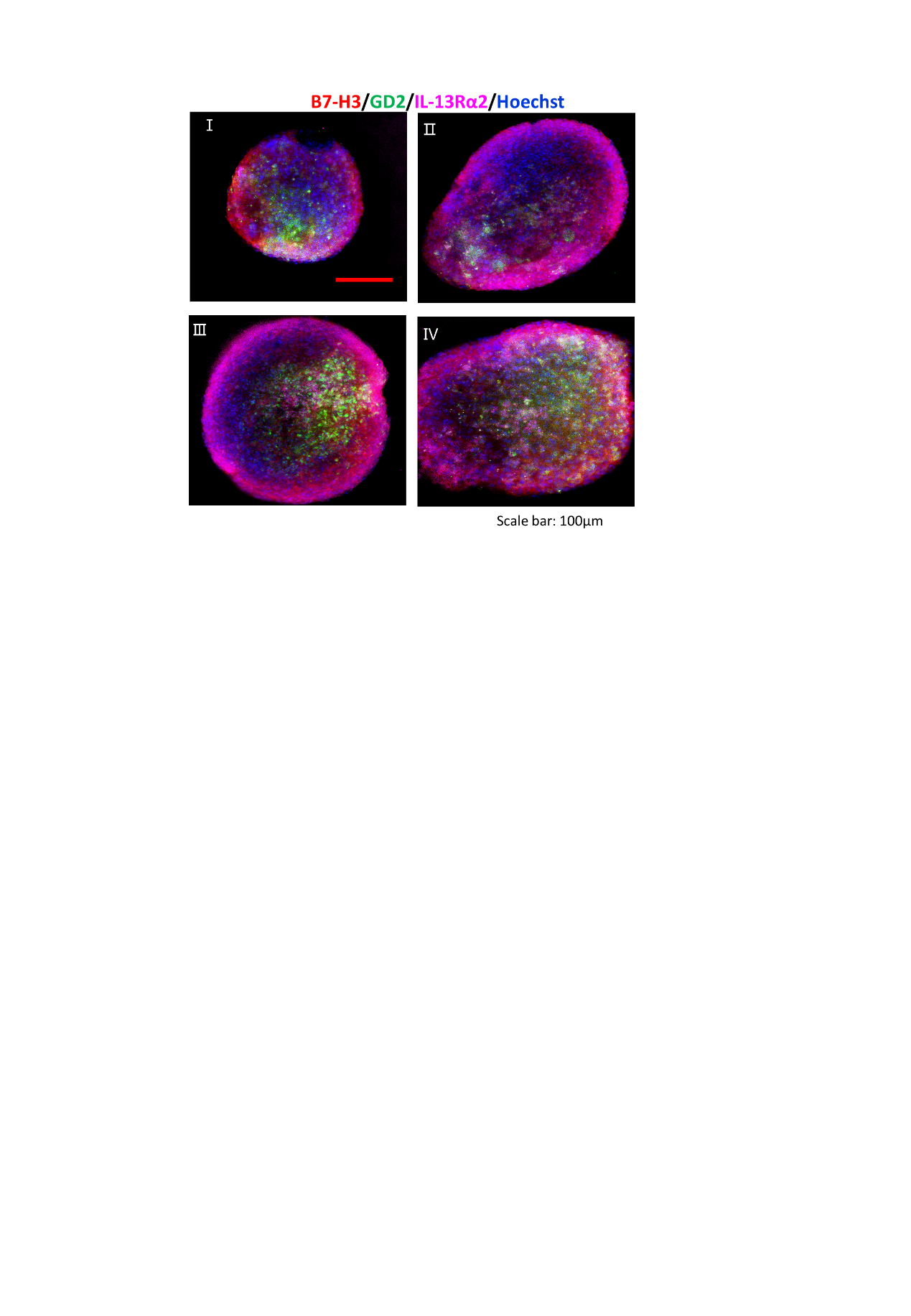


**Fig. S12. Heterogeneous of B7-H3, IL-13Rα2, and GD2 expression in different GBOs. Scale bar: 100 μm.**


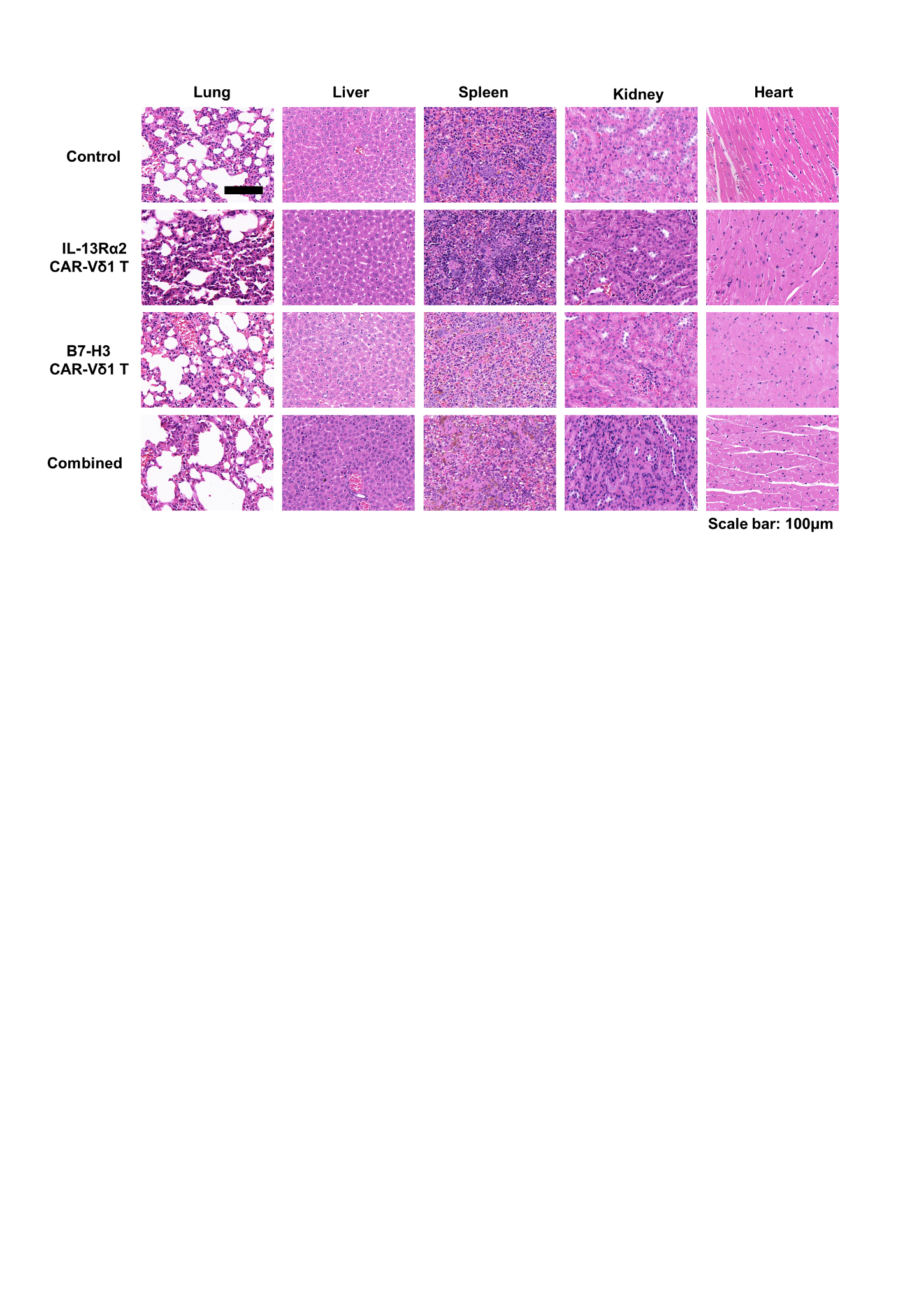


**Fig. S13. HE staining results of the heart, liver, spleen, lungs and kidneys of mice in different groups. Scale bar: 100 μm.**


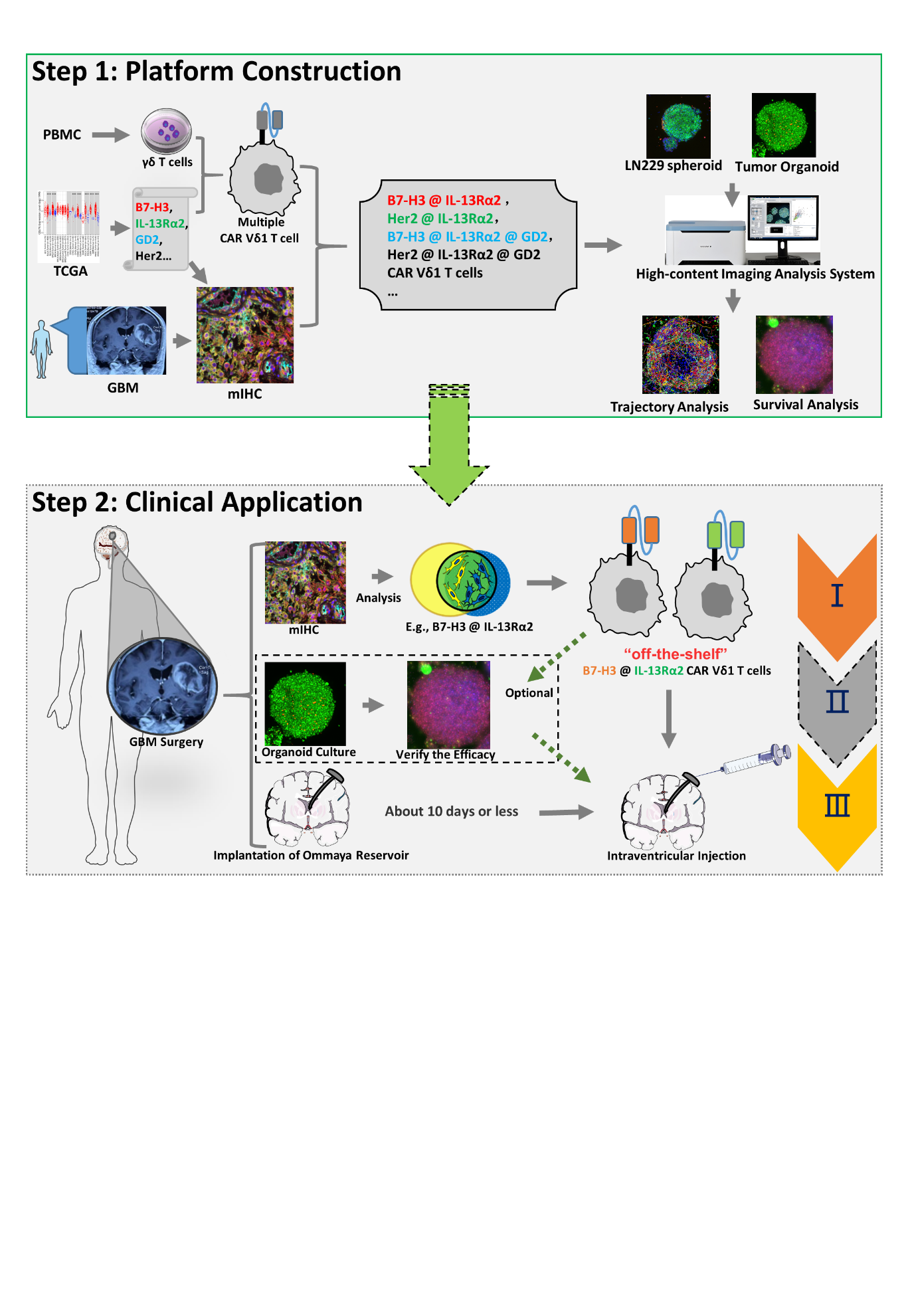


**Fig. S14. Experimental design flowchart and clinical application model diagram.**

**Supplementary Material**

**Table S1.** Targets used in preclinical studies and their characteristics(*39, 40*).

| **Target** | **Characteristics** |
| --- | --- |
| B7-H3 | - The immune checkpoint molecule binds to approximately 70% of neuroepithelial tumors(*41*). |
| IL-13Rα2 | - Present in more than 75% of GBMs(*42, 43*). |
| Her2 | - The EGFR family member HER-2, also known as ErbB2, is overexpressed in brain tumor cells. Receptor tyrosine kinase, which is overexpressed in 76% of primary GBM lines(*44, 45*). |
| GD2 | - GD2, also known as Disialoganglioside GD2, is highly expressed in GBM (over 50-80%)(*46, 47*). |
| CD133 | - CD133 is a marker of glioma stem cells, and its expression highly varies(*48*). |
| NKG2DL | - The expression of NKG2DL is upregulated in infected and malignant tumor cells(*49*). |
| CD70 | - The high expression rate of CD70 in glioblastoma and its significant expression in patients with poor prognosis make it a potential target for CAR-T cell therapy(*50*). |
| EphA2 | - EphA2 is highly expressed in over 60% of GBM tumor samples(*51*). |
| EGFRvIII | - The EGFR gene displays diverse mutations, including EGFRvIII, detected in around 30-50% of GBMs(*52, 53*). |

**Table S2.** General information and pathological diagnosis of the patients

| **Code** | **Gender** | **Age** | **Main diagnosis of admission** | **Pathological diagnosis** |
| --- | --- | --- | --- | --- |
| Patient 1 | Female | 32 | Malignant tumor of temporal lobe | Glioblastoma IDH wild type |
| Patient 2 | Male | 48 | Intracranial space occupying lesion | Glioblastoma IDH wild type |
| Patient 3 | Female | 65 | Malignant tumor of brain | Glioblastoma IDH wild type |
| Patient 4 | Male | 54 | Malignant tumor of frontal and temporal lobe | Glioblastoma IDH wild type |
| Patient 5 | Male | 62 | Malignant tumor of cerebrum | Glioblastoma IDH wild type |
| Patient 6 | Male | 67 | Malignant tumor of brain | Glioblastoma IDH wild type |
| Patient 7 | Female | 62 | Malignant tumor of temporal and occipital lobe | Glioblastoma IDH wild type |
| Patient 8 | Male | 55 | Malignant tumor of occipital lobe | Glioblastoma IDH wild type |
| Patient 9 | Male | 64 | Malignant tumor of brain | Glioblastoma IDH wild type |
| Patient 10 | Male | 42 | Malignant tumor of temporal lobe | Glioblastoma IDH wild type |
| Patient 11 | Male | 67 | Malignant tumor of parietal lobe | Glioblastoma IDH wild type |
| Patient 12 | Female | 58 | Malignant tumor of frontal lobe | Glioblastoma IDH wild type |
| Patient 13 | Male | 51 | Malignant tumor of frontal and parietal lobe | Glioblastoma IDH wild type |
| Patient 14 | Male | 58 | Malignant tumor of temporal lobe | Glioblastoma IDH wild type |
| Patient 15 | Male | 58 | Malignant tumor of parietal lobe | Glioblastoma IDH wild type |
| Patient 16 | Male | 68 | Malignant tumor of frontal and temporal lobe | Glioblastoma IDH wild type |
| Patient 17 | Female | 31 | Malignant tumor of frontal lobe | Glioblastoma IDH wild type |
| Patient 18 | Male | 50 | Malignant tumor of frontal lobe | Glioblastoma IDH wild type |

**Supplementary Material**

**Movie S1. A typical movie of B7-H3 CAR-Vδ1 T cells infiltrating and killing GBO.**

Blue: GBO (Hoechst pre-staining), Green: B7-H3 CAR-Vδ1 T cell (DIO pre-staining), Red: Dead cells (PI), Observation time: 15 hours, Laser channels: 405nm, 488nm, 561nm, Z-axis layer scanning: Height 200μm, Interval 10μm. Data acquisition: High Content Analysis CQ1 (Yokogawa, Japan), Processing software: CellPathfinder Software (Yokogawa, Japan).
